# Infrared nanospectroscopic mapping of a single metaphase chromosome

**DOI:** 10.1093/nar/gkz630

**Published:** 2019-07-25

**Authors:** Ewelina Lipiec, Francesco S Ruggeri, Carine Benadiba, Anna M Borkowska, Jan D Kobierski, Justyna Miszczyk, Bayden R Wood, Glen B Deacon, Andrzej Kulik, Giovanni Dietler, Wojciech M Kwiatek

**Affiliations:** 1 Institute of Nuclear Physics, Polish Academy of Sciences, PL-31342 Krakow, Poland; 2 Institute of Physics, Laboratory of Physics of Living Matter, Ecole Polytechnique Fédérale de Lausanne (EPFL), CH-1015 Lausanne, Switzerland; 3 Centre for Biospectroscopy and School of Chemistry, Monash University, 3800 Victoria, Australia; 4 Department of Chemistry, University of Cambridge, CB21EW, UK; 5 Department of Pharmaceutical Biophysics, Faculty of Pharmacy Jagiellonian University Medical College, PL-31007 Cracow, Poland; 6 School of Chemistry, Faculty of Science, Monash University, 3800 Victoria, Australia

## Abstract

The integrity of the chromatin structure is essential to every process occurring within eukaryotic nuclei. However, there are no reliable tools to decipher the molecular composition of metaphase chromosomes. Here, we have applied infrared nanospectroscopy (AFM-IR) to demonstrate molecular difference between eu- and heterochromatin and generate infrared maps of single metaphase chromosomes revealing detailed information on their molecular composition, with nanometric lateral spatial resolution. AFM-IR coupled with principal component analysis has confirmed that chromosome areas containing euchromatin and heterochromatin are distinguishable based on differences in the degree of methylation. AFM-IR distribution of eu- and heterochromatin was compared to standard fluorescent staining. We demonstrate the ability of our methodology to locate spatially the presence of anticancer drug sites in metaphase chromosomes and cellular nuclei. We show that the anticancer 'rule breaker' platinum compound [Pt[N(p-HC_6_F_4_)CH_2_]_2_py_2_] preferentially binds to heterochromatin, forming localized discrete foci due to condensation of DNA interacting with the drug. Given the importance of DNA methylation in the development of nearly all types of cancer, there is potential for infrared nanospectroscopy to be used to detect gene expression/suppression sites in the whole genome and to become an early screening tool for malignancy.

## INTRODUCTION

Heterochromatin and euchromatin are two distinct types of chromatin structure. Heterochromatin is a tightly packed form of DNA and has a condensed structure, containing ∼20% of the mapped human genome, which can be defined as transcriptionally inactive, but necessary for the maintenance of structural integrity (1–4). Euchromatin is the lightly packed form of DNA, which is the early replicating variety of chromatin containing most of the housekeeping genes (3,4).

One of the main chemical differences between eu- and heterochromatin is methylation of cytosine. DNA methylation determines gene expression, maintaining appropriate cellular functionality and genome integrity. DNA methylation affects activity of genes but also mechanical properties of DNA (5–8). These structural modifications may influence interactions between DNA and proteins (9) or other molecules such as drugs, dyes and the resistance to strand separation (10).

The technique of chromosome banding allows for the identification of individual chromosomes due to unique banding patterns on the specific regions of a chromosome. Chromatin organization is usually studied with light microscopy after the differential digestion of metaphase chromosome regions with chemicals or enzymes followed by staining with a dye. For example, G-banding, first developed in 1971 by Seabright (11), is still the most common cytogenetic method used to date (12) and consists of treating chromosomes with trypsin, to digest histones from the euchromatin followed by Giemsa staining. Darkly stained (G-dark) bands at fixed positions across the chromosomes represent regions of heterochromatin. The unstained or lightly stained areas (G-light bands) represent euchromatin. All the existing data on the spatial distribution of heterochromatin and euchromatin across chromosomes are difficult to interpret for two main reasons. First, the resolving power of an optical microscope (∼300 nm) is too low in order to locate precisely the heterochromatin–euchromatin boundaries, especially given the size of a human chromosome, which is between 2 and 10 μm. Second, metaphase chromosomes are highly compact thus the location of euchromatin and heterochromatin observed in the metaphase could differ from the chromatin locations found in extended interphase (13).

Human chromosome structure was also studied by microscopy techniques working beyond optical diffraction limit. Atomic force microscopy (AFM) identified thicker and thinner areas of chromosomes as dense heterochromatin and relaxed euchromatin respectively (14,15). Despite the nanometric spatial resolution achieved with AFM, the technique is not capable of delivering chemical and molecular information, thus the detection of G and R bands relies solely only on density/height. Label based super-resolution optical methods promise high resolution and strong signal intensities (16). Stochastic Optical Reconstruction Microscopy (STORM) was applied for *in situ* haplotype visualization using Oligopaint fluorescence *in situ* hybridization (FISH) probes in order to explore chromatin in single cells (17). Additionally, decondensed chromatin structure at transcription sites (active RNA pol II) was explored by binding activated localization microscopy with a spatial resolution up to 20 nm (18). Hu *et al.* employed Light-sheet Bayesian microscopy for imaging of photoactivable proteins on heterochromatin in fixed and living human cells (19), with a lateral resolution 50–60 nm (19). Although, super resolution optical methods deliver a limited amount of information on molecular structure. Moreover, fluorescence based methodologies require the introduction of labels, which may affect the delicate structure of the chromosome. Furthermore, fluorescence based measurements are also restricted by photobleaching, photoblinking or limited excited-state lifetimes and the lack of photon flux (20). Vibrational spectroscopy has also been applied to study chromosomes. Puppels *et al.* presented Raman spectra acquired from single chromosomes (21). This work and many following applications of Raman spectroscopy allowed for a label free investigation of chromosomes structure and composition (21–24). However, the diffraction limit did not allow for a detailed investigation into their structure at the nanoscale (22). Unstained human chromosomes have been analysed using a combination of backscatter imaging with a dark-field objective by applying visible and short near-infrared spectroscopy (25). This methodology enabled the size of the chromosomes, their refractive index gradients, the position of the centromers and also exploration more concentrated heterochromatin areas based on sample density to be determined (25). The main limitation of this approach is the lack of information about chemical composition of the chromosomes.

In contrast to all methods listed above, a combination of AFM and Infrared Spectroscopy (IR) allows one to obtain information about physical and chemical properties of the sample at very high spatial resolution without chemical labeling (26,27). This can be achieved by the application of two different techniques, measuring: i) the infrared light scattered by a scanning probe tip (26,28,29) (Fourier transform infrared nanospectroscopy, nano-FTIR) or ii) the response of AFM cantilever sensitivity to sample thermal expansion caused by an absorption of IR light (27) (phothermal induced resonance, AFM-IR). In our work, we used the second approach, since the photothermal detection has the strength to be purely related to the imaginary part of the IR light transmitted to the sample.

A number of biological systems have been investigated with AFM-IR such as single cells (30–32), bacteria (33,34), liquid-liquid phase separated protein droplets (35–37) and amyloid fibrils (26,38–42). The detection of low concentration chemical substances in cellular nuclei has also been demonstrated by localization of a rhenium-carbonyl complex after a 1-h incubation with 10 μl of an organometallic conjugate (31). Existing knowledge about chromosome structure and composition is very broad, but could be complemented and extended by the application of a nanoscale label-free analytical method. Here, we apply infrared nanospectroscopy (AFM-IR) to explore eu- and heterochromatin in single human metaphase chromosomes to achieve chemical spatial resolution below the diffraction limit. Using infrared nanospectroscopy, we study the methylation state at the sub-chromosome scale with a resolution approaching the order of 10 nm, enabling the chemical characterization of very small amounts of material. Our article describes the first nano-infrared study of investigation into metaphase chromosome structure and the first application of this technology to image anticancer drug binding sites to small biological samples such as chromosomes. Another very important aspect of our studies is direct visualization of DNA methylation, which role and influence on DNA structure still requires further research (9).

We have applied following systematic approach to generate eu- and heterochromatin nanoscale maps from single chromosomes: i) nanoscale spectra acquisition from single chromosomes and their comparison with standard micro and nanoscale spectra of cells, cellular nuclei and methylated, unmethylated DNA in order to explore nanospectroscopic markers of DNA methylation in chromosomes, ii) mapping of DNA methylation within chromosomes volume—CH_3_/OPO distribution, iii) an application of statistical analysis in order to fully explore nanoscale spectral difference between eu- and heterochromatin and verify/adjust the threshold on CH_3_/OPO distribution maps based on spatial location of spectra classified as eu- and heterochromatin, iv) a comparison of chromosome activity areas with areas of DNA methylation—a final proof of nanoscale detection of eu-and heterochromatic sites, v) an example of our methodology application – mapping of chemotherapeutic compound distribution in eu- and heterochromatin areas of single chromosome.

## MATERIALS AND METHODS

### Pt103 compound

Pt103 was synthetized by established method (43) and characterized by IR and F-NMR spectra (44).

### Cell culture

HeLa cells were grown in a humidified incubator at 37°C in 5% CO_2_ in complete growth medium composed of Dulbecco's Modified Eagle Medium (Life Technologies) containing 10% of inactivated fetal bovine serum (FBS) South American (Life Technologies) and 1% of Penicillin/Streptomycin solution (Life Technologies).

### Incubation with the Pt-103

For the drug detection experiment, cells were incubated with 50 μM of [Pt{N(p-HC_6_F_4_)CH_2_}_2_py_2_], for 4 h. Before supplying the culture medium (4 ml per flask) the appropriate amount of drug was dissolved in 20 μl of acetone. The same amount of pure acetone was added to the control samples.

### Chromosome preparation

Metaphase chromosomes were obtained from HeLa cells according to the procedure described in details in Supplementary Data S1.

### AFM-IR spectra collection and AFM-IR mapping

**Figure 1.**
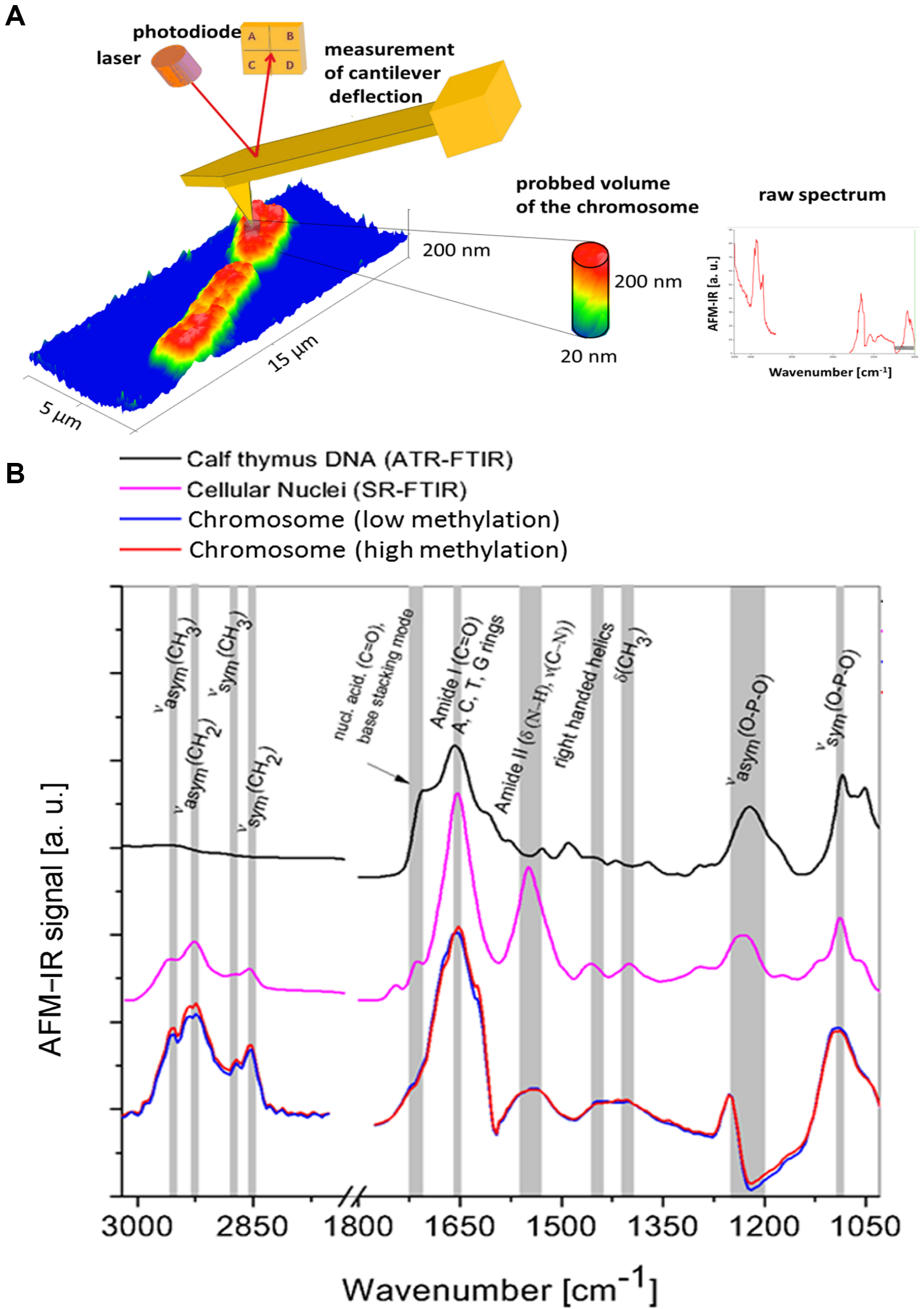
(**A**) a scheme of chromosome spectra acquisition via AFM-IR (**B**) A comparison of two kinds of AFM-IR spectra collected from three independent chromosomes (80 AFM-IR spectra were averaged for each spectrum kind: blue and red) with SR-FTIR a spectrum of cellular nuclei collected with the infrared microscope at the Australian Synchrotron (pink) along with a spectrum of ATR-FTIR spectrum of Calf thymus DNA (black).

**Figure 2.**
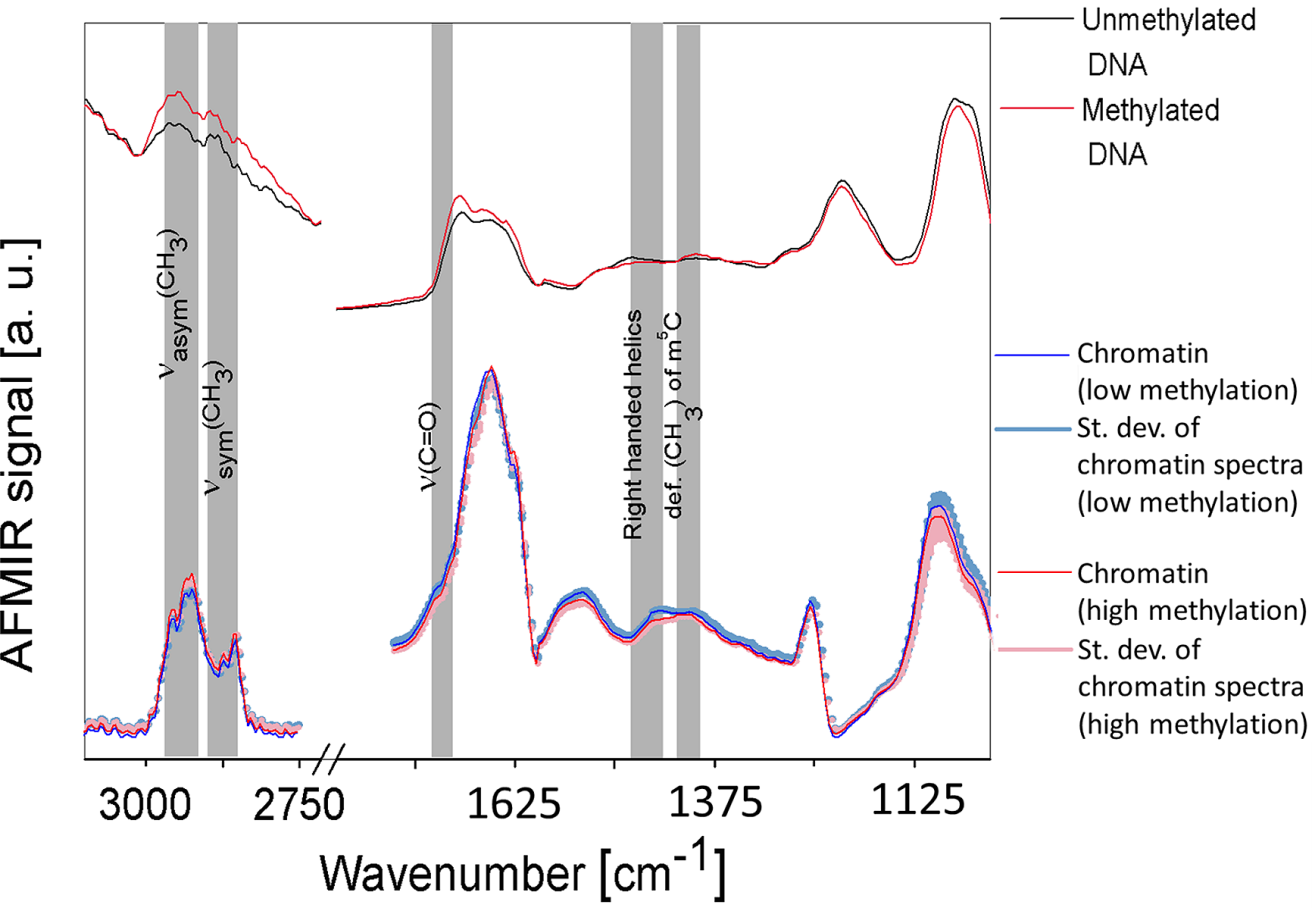
A comparison of AFM-IR infrared spectra collected from chromosomes, methylated DNA and unmethylated DNA, 80 averaged spectra collected from chromosome areas containing highly methylated chromatin, 80 averaged spectra collected from chromosome areas areas containing lowly methylated chromatin, 10 averaged spectra collected from methylated DNA and 10 averaged spectra collected from unmethylated DNA.

**Figure 3.**
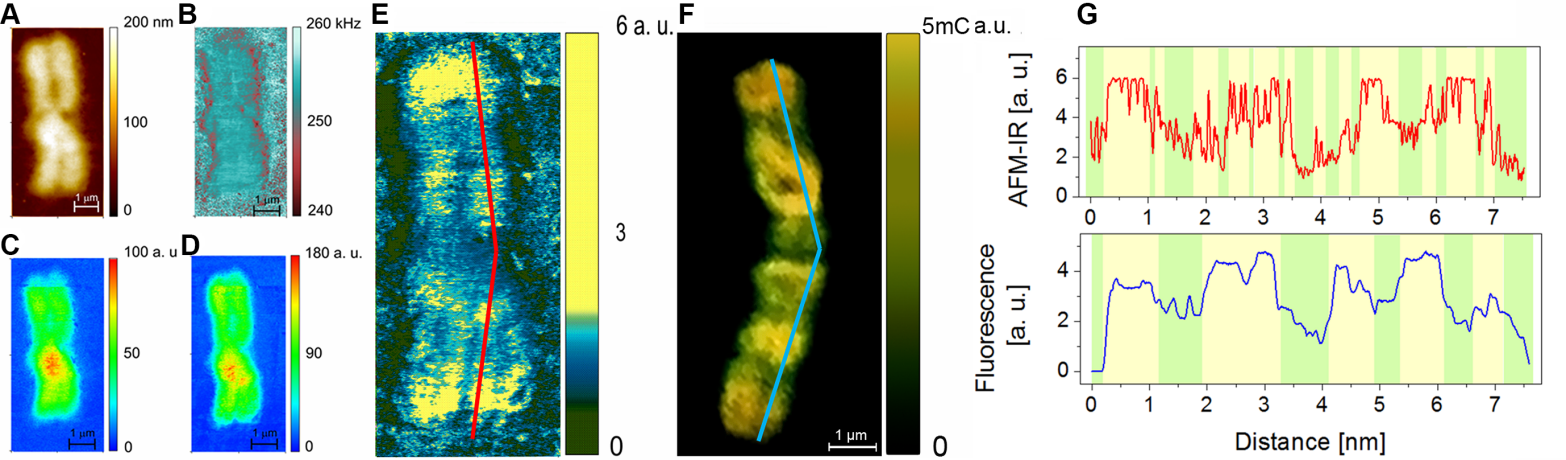
The AFM-IR results achieved for the typical human chromosome isolated from HeLa cell: (**A**) AFM topography, (**B**) the contact resonance frequency of AFM cantilever correlated to the local stiffness to demonstrate that the banding contrast is related with chemical structure not local stiffness heterogeneity, (**C**) the integrated area of an absorption band at 1240 cm^−1^ corresponding to the O-P-O asymmetric stretching vibration, (**D**) the integrated area of an absorption band at 2952 cm^−1^ corresponding to the vibration of methyl (-CH_3_) asymmetric stretching vibration, (**E**) the ratio of the integrated absorption band at 2952 cm^−1^ to the absorption band at 1240 cm^−1^, (**F**) anty 5-methylcytosine direct immunostaining counterstained with propidium iodide, similarities between (E) and (F) can be observed: high intensity of signal from 5- methylcytosine (F) correspond to high CH_3_/OPO ratio (E, yellow), (**G**) a direct comparison of profiles extracted along red (AFM-IR) and blue (fluorescence) lines; chromosome thickness: 190 – 210 nm, pixel size 7.8 × 7.8 nm.

**Figure 4.**
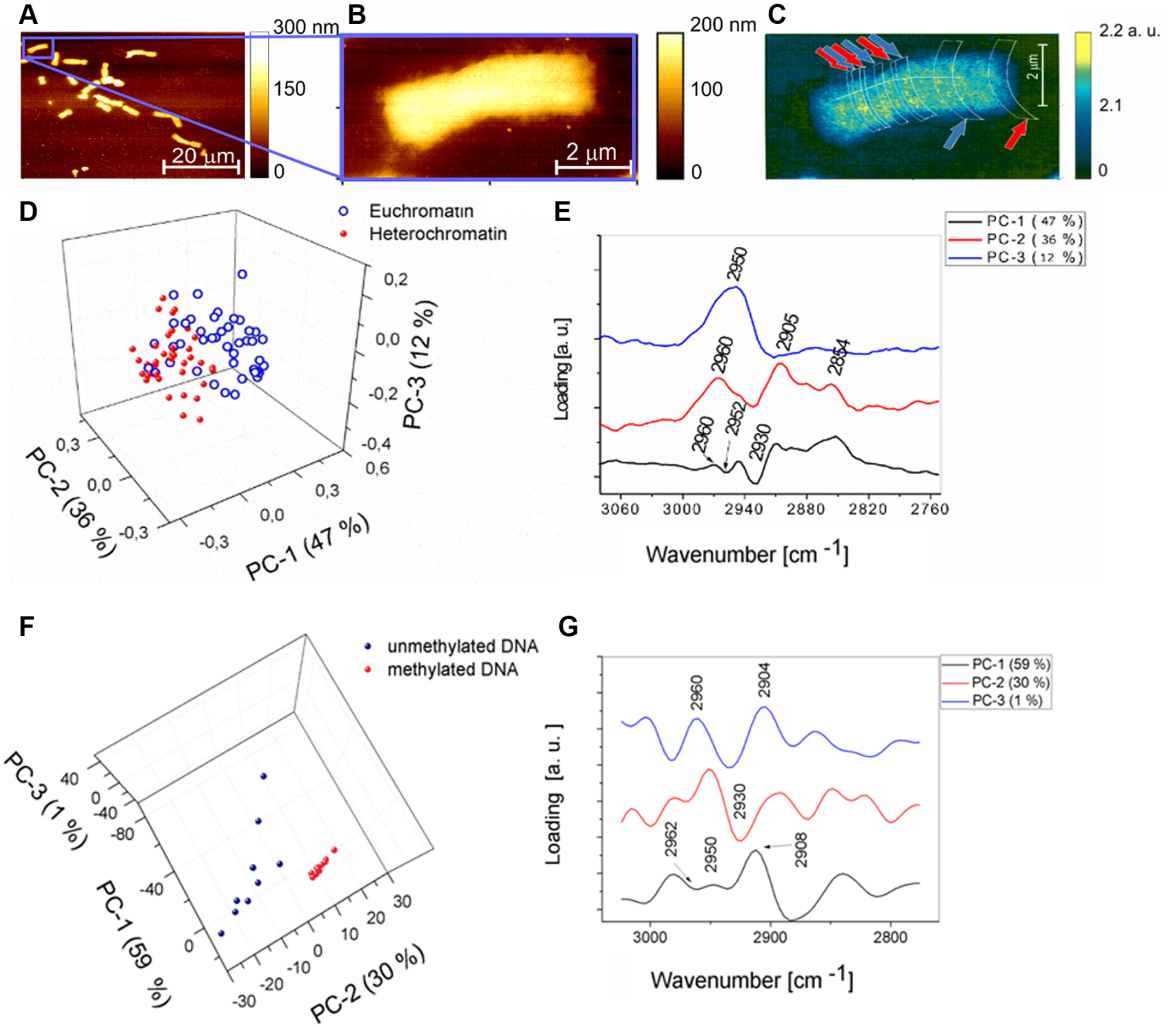
The results of PCA performed on spectra collected from a single chromosome and DNA in the spectral range from 3070 cm^−1^ to 2755 cm^−1^. (**A**) AFM topography of chromosomes isolated from HeLa cells, chromosome thickness 70–90 nm. (**B**) AFM topography of one chromosome isolated from HeLa cells. (**C**) The ratio of the absorption for the 2952 cm^−1^/1240 cm^−1^ bands with areas of the spectral collection marked by red arrows (heterochromatin) and blue arrows (euchromatin) where the spectra were recorded pixel size 6.4 × 6.4 nm. (**D**) Scores plot of spectra collected from a single chromosome presented in (C) showing scores projected onto PC-1, PC-2, PC-3. (**E**) Loadings plot corresponding showing loadings explaining the separation along PC-1, PC-2 and PC-3 presented in (D). (**F**) Scores plot of DNA spectra showing scores projected onto PC-1, PC-2, PC-3. (**G**) Loadings plot showing loadings explaining the separation along PC-1, PC-2 and PC-3 presented in (F).

**Figure 5.**
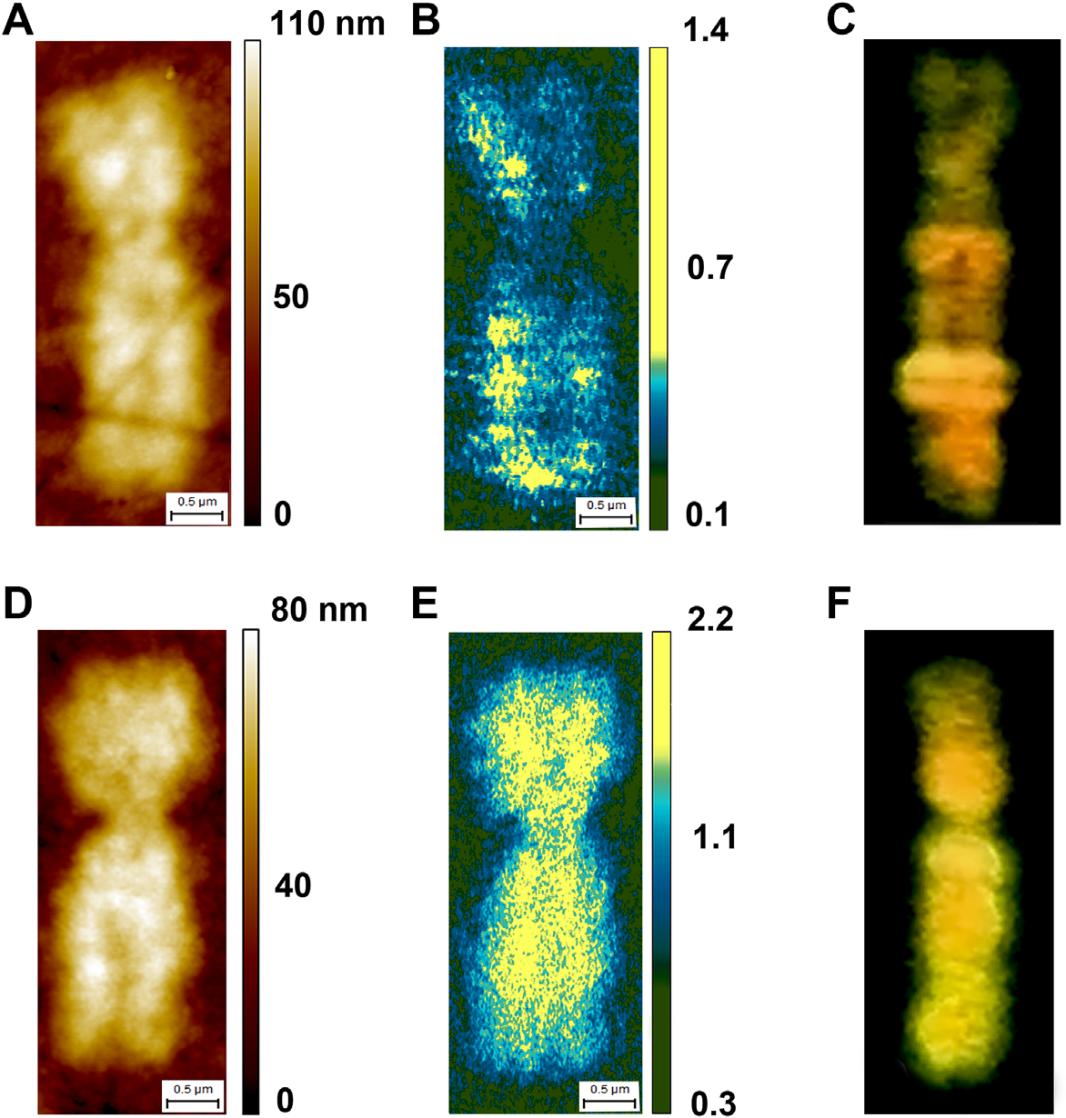
AFM-IR on Xs female chromosomes: (**A** and **D**) AFM topography of active X (A) and inactive—methylated X (D) female chromosome. (**B** and **E**) The ratio of the integrated absorption band at 2952 cm^−1^ to the absorption band at 1240 cm^−1^ of active X (B) and inactive X (E) female chromosome. (**C** and **F**) anty 5-methylcytosine direct immunostaining counterstained with propidium iodide of active X (C) and inactive X (F) female chromosome, similarities between B, C and E, F can be observed; chromosome thickness X_a_ 65 – 80 nm, X_i_ 60 – 80 nm, pixel sizes X_a_ 7.5 × 14 nm, X_i_ 6.2 × 14 nm.

**Figure 6.**
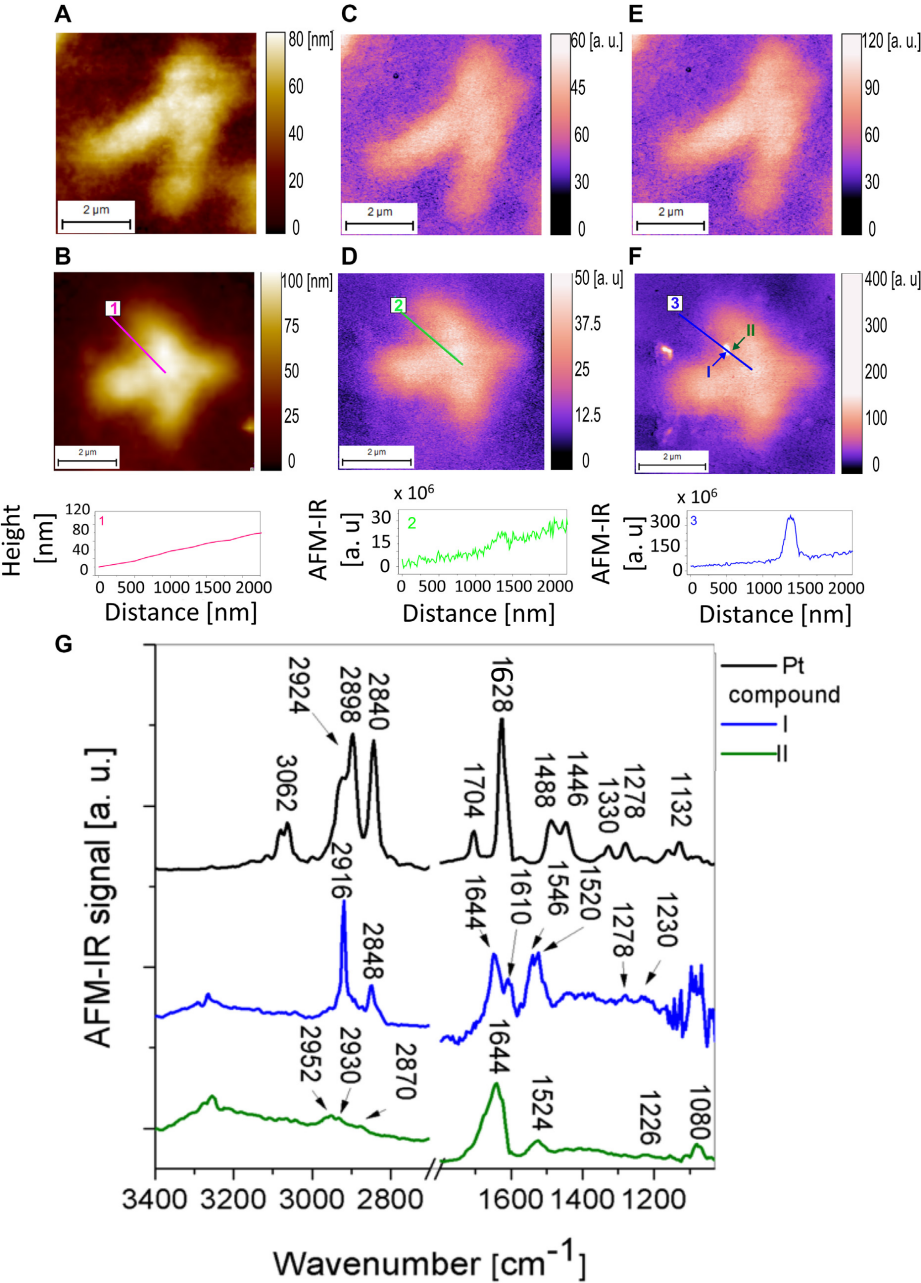
The detection of the Pt compound Pt103 interacting with the heterochromatin in single chromosome. (**A**) AFM topography of one chromosome isolated from control HeLa cell chromosome thickness 50–60 nm, pixel size 8 × 8 nm. (**B**) AFM topography of chromosomes isolated from HeLa cell treated with the platinum drug with the extracted height profile, chromosome thickness 55–70 nm, pixel size 10 × 10 nm. (**C**) The distribution of an absorption for the 1240 cm^−1^ band in control chromosome. (**D**) The distribution of an absorption for the 1240 cm^−1^ band in Pt-103 treated chromosome. (**E**) The absorption for the 2916 cm^−1^ band in control chromosome. (**F**) The absorption for the 2916 cm^−1^ band in Pt-103 treated chromosome with the extracted height profile. (**G**) Typical AFM-IR spectra collected from pure Pt compound and places marked in (F).

**Figure 7.**
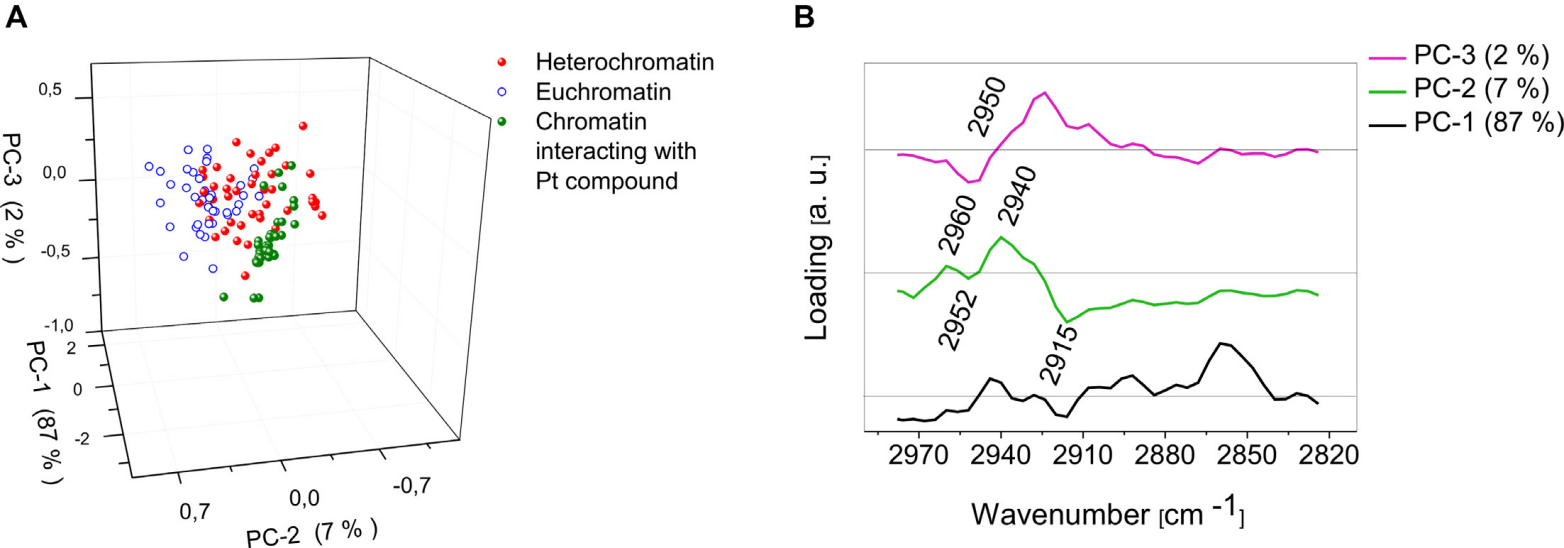
Classification spectra collected from chromatin interacting with the Pt-103, PCA analysis: (**A**) scores plot showing the clustering of spectra collected from eu- (blue) and heterochromatin (red) areas of single chromosome and spectra collected from chromatin interacting with the drug in eight various chromosomes (green) in the spectral range from 3070 cm^−1^ to 2755 cm^−1^, (**B**) loadings plot for A.

Spectra and maps were collected using two commercially available microscopes nanoIR and nanoIR2 (Anasys, Santa Barbara, CA, USA), both supplemented with a tunable infrared OPO (Optical Parametric Oscillator) lasers (Instrumental technical details are described in Supplementary Data S1). Results were obtained with nanoIR (Figures 1–7) and nanoIR2 (Figure 5). The first step in the measurement procedure was a generation of a topographical image of the metaphase chromosomes. Then, based on topography, chromosomes were chosen and the absorption of 1240 cm^−1^, 2952 cm^−1^ peaks corresponding to mainly phosphate groups from the DNA backbone and –CH_3_ groups were mapped. Pixel size is noted in each figure caption. All spectra were taken in the spectral range of 3600 cm^–1^ – 1000 cm^–1^ with 1024 scans co–added. Background of the OPO laser is presented and discussed in Supplementary Data S2. Details of data post-processing are described in Supplementary Data S1.

## RESULTS AND DISCUSSION

AFM-IR enables to map simultaneously the morphology and chemical properties of individual chromosomes at the nanoscale, by illuminating the sample with IR light at fixed wavenumber corresponding to a particular chemical bond vibration (Figure 1). Vertical and lateral resolution of the instrument are independent and they are defined by different physical constrains. In our work, to define effective lateral chemical resolution during imaging, we applied two standard methods. First, we demonstrated the sharpness of boundaries between eu- and heterochromatic areas in flat fragment of the chromosome and in small parts of the chromosome called double minutes (∼60 nm thickness). Then, we used as model system amyloid fibrillar aggregates, with nanoscale dimensions, to probe the closest distance at which two different objects can be still distinguished. Our measurements indicate that the effective lateral resolution ranges approximately between 15 − 40 ± 20 nm on chromosomes and double minutes and between 10–30 nm for amyloid fibrils. This relatively broad range and high value of the uncertainty are determined by scanning and sampling rates and are related to several factors affecting the resolution such as the tip apex size and thermomechanical properties of the investigated sample (45–50). In addition, our large error derives from the consideration of divisions maps. Details are presented in Supplementary Data S3. The vertical resolution is defined by the minimal detectable thermomechanical expansion and it has been demonstrated to be in the order of 30–50 nm for bottom illumination and few nanometers for top illumination of the sample by the laser (38). Furthermore is necessary to consider the depth resolution of the instrument that is significantly larger than the effective vertical and lateral spatial resolution. The depth resolution is strictly dependent on the indentation depth of the field inducing thermal expansion of the sample. An evanescent field or infrared wave extends through the sample film to a distance on the order of the wavelength of light which is equal to micrometres for the mid infrared spectral range (27). Therefore the evanescent field (nanoIR) and infrared beam (nanoIR2) pass through entire thickness of the chromosomes (80–200 nm, Figure 1), which is at least one order of magnitude smaller than the order of the wavelength of infrared light (3–10 μm).

### AFM-IR band assignment of single metaphase chromosome spectra

The lateral spatial resolution that could be achieved using SR-FTIR is ∼2.5 μm, (wavelength dependent) (51), making it impossible to record individual spectra of single chromosomes. AFM-IR spectra of the chromosome were compared with spectra of cellular nuclei and DNA collected by conventional FTIR methods to assess whether they are comparable with the AFM-IR spectra. Figure 1 shows a comparison of: (i) AFM-IR spectra (80 averaged spectra for each of two slightly different types of spectra collected from chromosomes, 160 in total) recorded in the regions of three chromosomes (from three various samples) with each spectrum collected using a synchrotron light source: (ii) a spectrum of cellular nuclei collected in transmission mode with the infrared microscope at the Australian Synchrotron, and a spectrum collected using FTIR spectroscopy: (iii) ATR-FTIR (Attenuated Total Reflectance—Fourier Transform Infrared spectroscopy) spectrum of Calf thymus DNA. As expected, the chromosome spectrum is an equivalent of a convolution of the cellular nuclei spectrum and the spectrum of calf thymus DNA, therefore artifacts related to optical effects such as scattering were not observed. Each spectral feature typical for DNA and cellular nuclei are observed in the chromosome spectra including the symmetric and asymmetric stretching bands of phosphate from the DNA backbone at 1088 cm^−1^ and 1240 cm^−1^, respectively (52). Amide I and Amide II bands from histones appear at 1654 cm^−1^ and 1549 cm^−1^, respectively (53–55) and the symmetric and asymmetric stretching of methyl and methylene vibrations appear in the spectral range from 3960 cm^−1^ to 2850 cm^−1^ (52,53). Detailed band assignments are listed in Supplementary Table S1 (Supplementary Data S4). Differences between two described groups of spectra were observed mainly in bands related to methyl and methylene motions such as ν_sym_(CH_2_), ν_sym_(CH_3_)_,_ ν_asym_(CH_2_), ν_asym_(CH_3_) and in the intensity of the band assigned to right-handed helices at 1440 cm^−1^ (52,53). Direct comparison of chromosome spectra collected at the nano and micro scale could not be performed because single chromosomes are too small to achieve the sufficient signal-to-noise ratio required when using ATR-FTIR or SR-FTIR spectroscopy. Furthermore, the sample preparation procedure does not allow for preparation of bulk samples containing only chromosomes for infrared spectroscopy because chromosomes are released upon contact with the surface as described in the methods section and despite Colcemid treatment, only around 5–15% of cells dependent on the donor's health, age, conditions etc. are arrested at metaphase (56,57).

### The origin of differences in the methylation degree along single metaphase chromosome

To prove further that the technique is able to distinguish between methylated and unmethylated sites in a DNA in chromatin, AFM-IR spectra acquired from metaphase chromosomes were compared with the AFM-IR spectra of commercially available methylated and unmethylated DNA. Figure 2 shows a direct comparison of the average of AFM-IR spectra typical for lower methylation state (80 spectra) and the chromatin areas with a slightly higher level of methylation (80 spectra), together with their standard deviation. These spectra are compared to AFM-IR spectra collected from methylated and unmethylated DNA, where each spectrum is the average of 10 independent measurements. Highly overlapping bands were further resolved by calculating the second derivatives (Supplementary Data S5 and Figure S6) in the spectral regions of interest including: (i) methyl and methylene stretching between 3100 cm^−1^ – 2750 cm^−1^ (Supplementary Figure S6b) (58–60) and (ii) between 1520 cm^−1^ and 1300 cm^−1^ (Supplementary Figure S6c) (54,55). In the first type of spectra (red color) an increase in the intensity of the asymmetric stretching vibration of the methylene and methyl motions at 2927 cm^−1^ and 2960 cm^−1^ - 2952 cm^−1^ is observed. In addition, there is also an increase in intensity in the methyl deformation mode from cytosine at 1408 cm^−1^ (52,53). These spectral changes were also observed in the spectra of the methylated DNA but were absent in unmethylated DNA; indicating the changes come directly from the nucleic acids and not the surrounding histones. According to previous studies (52) methylation affects DNA structure, which could be detected as changes in the infrared spectral range from 1440 cm^−1^ to 1360 cm^−1^ (52). The second type of spectra (blue) show an increase in the intensity of the C = O stretching mode at 1723 cm^−1^, which is possibly related to the histone acetylation. This finding is consistent with the literature, which reports that hyperacetylation of the histone tails is typical for less methylated euchromatin (61). There is also a decrease in the intensity of the band assigned to the right-handed helices at 1440 cm^−1^ (52,53) in the spectra collected from more methylated chromatin. Similar spectral changes were observed in pure methylated DNA compared to unmethylated DNA, as previously reported by Banyay and Graslund, who determined specific infrared markers for various degrees of methylation in d(CCGGCGCCGG)2 (52).

### Nanospectroscopic mapping of DNA methylated sites in a single metaphase chromosome

The next step of an investigation into metaphase chromosome structure was mapping of the distribution of methylated sites along a single chromosome. The instrumental setup enabled us to generate a map for a discrete wavenumber value corresponding to a particular vibration in the area of interest. The resultant maps show the IR absorption peak height at a given wavenumber for each pixel. Figure 3 displays the results achieved for a typical human chromosome isolated from HeLa cells including the AFM topography (Figure 3A), the contact resonance frequency of the AFM cantilever (Figure 3B) showing the distribution of mechanical properties of the sample, an IR absorption map at 1240 cm^−1^ corresponding to the O-P-O asymmetric stretching mode of dry DNA, being mainly in the A-DNA form (62,63) (Figure 3C) and an IR absorption map at 2952 cm^−1^ corresponding to the vibration of the methyl asymmetric stretching mode (Figure 3D). Methyl and methylene motions are considered as potential markers for different chromatin types because of the density of methylated purines and pyrimidines, mainly cytosine, which density increases from the euchromatin toward the heterochromatin (64). Figure 3D shows methyl group distribution, however, the banding pattern is not visible because this image is affected by the material density/height. To avoid the influence of chromosome thickness directly related to the amount of material, the signal collected from the distribution of methyl groups at 2952 cm^−1^ (Figure 3D) was divided by the distribution of phosphate groups at 1240 cm^−1^ (Figure 3C), corresponding to the DNA density, which is directly related to the density/thickness of the chromosomes. The ratio depicted in the Figure 3E shows the banding pattern, yellow areas contain more methylated sites of chromatin and green areas contain less methylation. In Figure 3E, the pure chemical information from DNA methylation, which is not affected by sample thickness/density within the chromosome, is clearly shown.

The O-P-O asymmetric stretching vibration from the DNA backbone at 1240 cm^−1^ was chosen for normalization despite the low IR signal intensity in the spectral range from 1230 cm^−1^ – 1190 cm^−1^, which is related to low laser power in this spectral range (discussed in Supplementary Data S2). The normalization to the AFM topography (division by topography) would not be an appropriate method because of the heterogeneity of our relatively complex biological sample (14,27,65).

To observe the banding pattern of chromosomes independently direct immunofluorescence staining of 5-methylcytosines (5mC) in DNA was performed using the Imprint® Monoclonal Anti-5-methylcytosine antibody produced in mice (Sigma-Aldrich) and counterstained with propidium iodide (Sigma-Aldrich) for better contrast (Figure 3F) as described in detail in Supplementary Data S6 (66,67). Based on the precise calculations of ratios of chromosome p and q arms, all chromosomes from a particular metaphase were classified and ordered in a karyogram (Supplementary Data S6 and Figure S7). The ratio is typical for each individual chromosome. Therefore, it was possible to recognize the chromosome mapped by AFM-IR as chromosome 2 based on a comparison with another chromosome 2 analyzed by immunofluorescence. As expected, a similar distribution in the methylation-banding pattern was observed in both chromosomes as shown in Figure 3E and F. The high intensity of signal from 5-methylcytosine (Figure 3F) corresponds to high CH_3_/OPO ratio (Figure 3E, yellow). For a more quantitative comparison of spectroscopic and fluorescence data we are presenting profiles extracted from AFM-IR and fluorescence images along lines marked on both maps. Highly methylated areas are highlighted in yellow. This direct comparison proved that highly methylated areas could be better resolved with AFM-IR compared to fluorescence microscopy. It is known, that the proportion of active and inactive chromatin is likely to be preserved in metaphase chromosomes (68). However, line profiles extracted along the two chromosomes do not ideally coincide with each other. The observed inhomogeneity is related to the fact that both chromosomes have been extracted from different cells, which are likely to be in slightly different stages of metaphase. Even if both chromosomes are in metaphase, the chromatin condensation and the size (length and thickness) of chromosomes, which determines the location of eu- and heterochromatic areas, is likely to be slightly different (69,70). Additionally, these two cells could have slightly different metabolic activity and indeed different eu-/heterochromatin content (66,67,71). Upon the deposition on the substrate the chromosome arms may also bend and therefore the eu- and heterochromatin areas observed in the profiles could be shifted relative to one another. Moreover, fluorescence microscopy is limited because DNA bases including 5mC modified bases are hidden within the double-stranded DNA helix (72). AFM-IR spectroscopy can detect the degree of methylation from the 3D volume of the sample under the AFM tip (73) as schematically presented in Figure 1. Therefore, AFM-IR provides information from the area of the chromosome, which could be unavailable using fluorescence probes. These factors cause the inhomogeneity observed between results obtained with AFM-IR and fluorescence microscopy.

Principal component analysis (PCA) analysis was applied to reduce the dimensionality of the data set and to resolve hidden spectral differences between eu- and heterochromatin. The main results of PCA are the scores plots and loadings plots. Original variables—spectra are projected in the space of new variables called principal components PCs, which are based on linear combinations of the original variables. Scores plots project each spectrum as a single point on a new coordinate system, defined by principal components. Each PC explains a percentage of the total variance within the data set (PC1>PC-2>…>PCn). Therefore, only several first PCs are taken into consideration. Loadings plots show which bands are varying the most and are responsible for the clustering observable in the scores plots. In our studies several PCA models described in the next paragraphs were tested.

For the first model of PCA the spectra acquired from the single metaphase chromosome presented in Figure 4A–C, were chosen, because areas containing heterochromatin are very small and the banding pattern is not that easily visualized in the infrared maps (Figure 4C). However, it was possible to select the color threshold in order to visualise the eu- and heterochromatin areas. In order to verify further that the spectral differences observed between the two chromosome areas containing mainly heterochromatin or euchromatin are related to methylated and unmethylated deoxyribonucleic acid PCA was performed on AFM-IR spectra collected from methylated and unmethylated DNA. In total four PCA models were calculated for spectra acquired from both DNA and chromosomes: (i) in the finger print region: 1750 cm^−1^—1230 cm^−1^ and (ii) in the methyl and methylene stretching region 3070 cm^−1^–2755 cm^−1^. The most definitive separation was related to the level of methylation as shown in the methyl and methylene stretching spectral region 3070 cm^−1^–2755 cm^−1^. The data is presented in Figure 4 and the results of the PCA in fingerprint spectral range 1750 cm^−1^–1230 cm^−1^ are shown and discussed in Supplementary Data S7 and 8.

Figure 4D demonstrates a separation of spectra into two groups collected from areas containing (i) heterochromatin (marked by red arrows on Figure 4C) and (ii) euchromatin (marked by blue arrows on Figure 4C). The separation along PC-1, explains 47% of the total variance within the dataset. Spectra collected from euchromatin are clustered in positive PC-1 space and all positive bands presented on the loading plot (Figure 4E) of PC-1 are typical for these spectra. Spectra collected from the heterochromatin are clustered in negative PC-1 space, which suggests that all negative bands in the PC-1 loadings plot are characteristic for heterochromatin. The loadings plot demonstrates that PC-1 is positively correlated with the CH_3_ asymmetric stretching vibration of the methyl group at 2960 cm^−1^ (spectra in positive PC-1 space - euchromatin) and it is negatively correlated with the same group at 2952 cm^−1^ (spectra in negative PC-1 space—heterochromatin). The results indicate a different position of the CH_3_ motion in the AFM-IR spectra collected from these two groups of spectra, and suggest that one of these two varieties of chromatin has CH_3_ groups in slightly different chemical environments. The PC-2 loadings plot (Figure 4C, red line) explains the separation along PC-2 (35% of total variance). This loading is dominated by CH_3_ symmetric and asymmetric stretching at 2905 cm^−1^ and 2960 cm^−1^, respectively. The symmetric stretching of the CH_2_ group at 2854 cm^−1^ is also an important loading that discerns the two groupings along PC-2. A separation along PC-3, which accounts for 12% of total variance, is dominated by a band assigned to the CH_3_ asymmetric stretching vibration at 2950 cm^−1^.

Second model of PCA was performed on AFM-IR point spectra of DNA (Figure 4F and G) in the methyl and methylene stretching region. This PCA model confirmed that the degree of DNA methylation can be detected by infrared absorption. Characteristic features responsible for clustering of methylated and unmethylated DNA spectra (Figure 4G) are very similar to spectral features responsible for the clustering of spectra collected from hetero- and euchromatin (Figure 4E). PC-1 is positively correlated with the CH_3_ asymmetric stretching vibration at 2950 cm^−1^ (methylated DNA) and negatively with the same band at 2962 cm^−1^ (unmethylated DNA). This result indicates a different chemical environment/position of the CH_3_ group in methylated DNA compared with unmethylated DNA. Separation along PC-2 (unmethylated positive, methylated negative) is dominated by asymmetric stretching of CH_3_ at 2950 cm^−1^ (methylated DNA) and symmetric stretching of CH_3_ at 2890 cm^−1^ (unmethylated DNA). PC-3 is dominated by the asymmetric stretching vibration of CH_3_ at 2960 cm^−1^ and the symmetric stretching vibration of CH_3_ at 2880 cm^−1^.

In order to distinguish eu- from heterochromatin it is sufficient to use only the first PC (separation along PC-1). However, to explore fully spectral differences within the data set three PCs: PC-1, PC-2 and PC-3 should be taken into consideration. The application of PCA was indispensable to verify that the CH_3_/OPO ratio maps reflected the real distribution of hetero- and euchromatin along the chromosome. The location of each single spectrum classified by PCA as eu- or heterochromatin was carefully correlated with the CH_3_/OPO ratio map. Therefore, we can unequivocally verify the eu-/heterochromatin areas by: (i) mapping the density of low/high methylene distribution and (ii) statistical confirmation that the collected spectra were indeed classified correctly as eu-/heterochromatin. The threshold of OPO/CH3 maps was different for each chromosome (Figures 3–5 and Supplementary Figure S11) and selected carefully in order to demonstrate the banding patterns and completed by PCA results, details can be found in Supplementary Data S1.

### A comparison of metaphase chromosome methylation level and chromatin activity

The chromatin activity can be directly observed by comparing of the distribution of methylated DNA in both Xs of active and inactive female chromosomes (Figure 5). In order to prove the ability of AFM-IR to follow active (unmethylated DNA) and inactive (methylated DNA) chromatin, two single metaphase X female chromosomes: one inactive (fully methylated DNA) and the second one, active (containing eu- and heterochromatin areas, forming a characteristic banding pattern) were investigated. Both metaphase X female chromosomes analysed were extracted from the same metaphase cell. A distribution map of methylation (CH_3_/O-P-O ratio) in the two X chromosomes is shown in Figure 5b,e. The results have been compiled based on the distribution of DNA methylation characterized by 5-methylcytosines (Figure 5C and F).

Additionally, to confirm the quality of the obtained results, and the more detailed information delivered with respect to previously described methods FISH was applied using an Alu probe, which is described in detail in the Supplementary Data S9 (74,75). The total amounts of chromatin, heterochromatin and euchromatin were precisely calculated in each single chromosome of the entire human genome using DNA sequencing (76,77). The results provided by The Human Genome Sequencing Consortium are consistent with the amounts of the particular type of chromatin, which was detected by infrared nanospectroscopy. By integrating all of the color pixels attributed to heterochromatin, we have calculated the percentage of eu- and heterochromatin in each mapped chromosome and we compared with corresponding values in the literature data, see detailed calculations in Supplementary Data S10. The total amount of heterochromatin varies from 2.13 to 52.15% for chromosome 2 and Xi (inactive X chromosome), respectively. The results for chromosome 2 and other chromosomes (Supplementary Data S10) are slightly above the values presented in the literature (76,77). The ratio between the two states of chromatin in each cell depends on many criteria including: type and stage of the cell (growth phase), inter-individual variations between donors, probability of unknown disease etc. Therefore, differences between the amount of heterochromatin obtained from AFM-IR maps and the literature data (77,78) (averaged data provided by International Human Genome Sequencing Consortium) are slightly different. The inactive X chromosome was not sequenced because it does not contain active DNA (76,77,79). Literature is lacking information on the percentage of methylated DNA in inactive X chromosomes and therefore the result obtained using AFM-IR cannot be compared to any other technique. The slight excess is probably related to the high sensitivity of AFM-IR to histone methylation. A good agreement between AFM-IR and literature data has confirmed the appropriate selection of color (intensity) threshold when calculating the AFM-IR maps using the (CH_3_/OPO) ratio. The threshold adjustment is strictly related with statistical analysis and discussed in next paragraph.

Direct comparison of the active and inactive X chromosomes constitutes the most important outcome of this research, which is definitive proof that infrared nanospectroscopy mapping of inactive heterochromatin and active euchromatin areas in metaphase chromosomes is indeed possible. All of the results presented above confirmed that we were able to map the high and low methylated areas of the chromosome. Here we have proven that AFM-IR mapping supported with appropriate data analysis is able to differentiate between active and inactive X chromosomes derived from the same metaphase cell.

### Mapping of platinum anticancer drug distribution in a single metaphase chromosome

Vibrational spectroscopy can play a significant role in studies of the interaction between chemotherapeutic drugs such as metallo-drugs and single cells. This approach enables the monitoring of the biochemical impact and physiological reaction of cells to anticancer agents (80,81). Here, we show that AFM-IR spectroscopy coupled with PCA can identify which type of chromatin is more sensitive to the binding of a Pt anticancer drug.

The methodology is exemplified by the ‘rule breaker’ platinum anticancer drug, [Pt{N(p-HC_6_F_4_)CH_2_}_2_py_2_] Pt-103, which is a four coordinate platinum(II) complex. This compound does not have hydrogen substituents on the donor nitrogen atoms (81). The drug already has shown anticancer activity against cisplatin-resistant cell lines and is active *in vivo* (81,82). Pt-103 has caused nearly complete tumor regression in the ADJ/PC6 mouse tumor model (83). This very high cytotoxicity, and enhanced cellular uptake in cisplatin resistant cells (83) is related to greater hydrophobicity of Pt103 in comparison to cisplatin (81,84). Under physiological conditions (pH ∼7.8) prior to interaction with DNA Pt-103 loses pyridine rings or more likely {N(p-HC_6_F_4_)CH_2_}^2-^ upon nucleophilic attack. In our recent studies, we applied X-ray emission spectroscopy and multiscale molecular dynamics calculation in order to test those two possible pathways of hydrolysis in the drug dissolved in aqueous buffer solution. X-ray studies confirmed that more likely the {N(p-HC_6_F_4_)CH_2_}^2-^ moiety acts as the leaving group and then [Pt(Py_2_)]^2+^ ligand interacts with adenine rather than guanine (85). However the dissociation of pyridine is also possible (85).

Here, the accumulation and location of the drug (discrete foci) was detected by AFM-IR and the type of chromatin in these areas was explored by applying PCA to spectra collected from the areas neighbouring the binding places of drug. Considering the lateral spatial resolution calculated in Supplementary Data S3 the location of the drug foci can be determined with nanometric precision (15–40 nm) in the plane of the sample. The location of the drug foci in z direction can not be determined because the signal was acquired from entire thickness of the chromosome (Figure 1).

HeLa cells were incubated with 50 μM of Pt- 103 for 4 h prior to chromosome isolation. AFM topographies of single investigated chromosomes isolated from control cells and cells treated with Pt-103 are shown in Figure 6A and B, respectively. The AFM-topographical images showed no differences between control and chromosomes treated with the anticancer drug. Furthermore, an IR absorption map of the band assigned to the asymmetric phosphodiester stretching vibration at 1240 cm^−1^ does not indicate any differences between them (Figure 6C and D). However, in the chromosomes isolated from the cells treated with the drug, there were small areas with a strong absorption band at 2916 cm^−1^ (H-C-H asymmetric stretching motions) in comparison with untreated chromosomes (Figure 6E and F). A strong band related to H-C-H asymmetric stretching vibrations was also observed at 2898 cm^−1^ (86) in the pure drug spectrum (Figure 6g). One can compare profiles extracted along the lines marked in the AFM-IR maps showing the distribution of the 1240 cm^−1^ and 2916 cm^−1^ bands (Figure 6E and F). In each of the 20 investigated chromosomes isolated from the cells incubated with the drug, this very strong absorption at 2916 cm^−1^ was observed in multiple instances. Drug foci were observed in 65–75% of the metaphase chromosomes. For each chromosome 1–4 foci were detected.

The experiment was repeated in triplicate.

Reproducible spectra were collected from the places indicating very strong absorption at 2916 cm^−1^. In Figure 6G one of the AFM-IR spectra (blue spectrum) is presented and compared with a spectrum collected very close to the region of the drug binding (green spectrum) in the same type of chromatin. The regions within the chromosome marked by arrows are where the spectra were recorded (Figure 6F). The AFM-IR spectrum of pure Pt-103 is also presented in Figure 6G for comparison. To assign the vibrational modes in the spectrum of the Pt-103 and the spectrum collected from the chromosome treated with the drug, we calculated the theoretical IR spectra of the pure Pt-103 molecule. Calculations (Supplementary Data S11) were done in the liquid phase. The CH_2_ asymmetric and symmetric stretching vibrations are observed in the AFM-IR spectrum of the pure drug at 2898 cm^−1^ and 2840 cm^−1^, respectively, and in the spectrum of the drug already bonded to the DNA at 2916 cm^−1^ and 2844 cm^−1^. The presence of these peaks confirms that with the AFM-IR spectroscopy we have detected Pt-103 molecules inside the chromosomes. The drug influence was also detected in the fingerprint spectral region. The most significant marker of the Pt-103 molecule was an intense peak at 1628 cm^−1^ from the tetrafluorophenyl ring breathing mode (aromatic C = C stretching) in the pure drug. A relatively low intensity band from the tetrafluorophenyl ring breathing was detected at 1610 cm^−1^ in the spectra from chromosomes isolated from the cell treated with Pt-103. Summarizing, our experimental data supported by theoretical calculations confirms the existence of the tetrafluorophenyl ring and the (-CH_2_-CH_2_-) group within the chromosome volume as a signature of the Pt-103 compound. Our previous X-ray scattering studies (85) unveiled a hydrolysis process of Pt-103 and binding nature to DNA (preferentially adenine).

Visualization of the subcellular distribution of anticancer compounds yields unique, valuable information on the location of the sites relevant to their biological activity. Infrared nanospectroscopy was applied also to demonstrate the drug distribution in isolated cellular nuclei, with nano-metric spatial resolution. HeLa cells were incubated with 50 μM of Pt-103 for 4 h and then cellular nuclei were isolated according to the procedure described previously by Lipiec *et al.* (87). Detailed results are presented in Supplementary Data S12. Obtained data indicated an accumulation of Pt-103 discrete foci in the chromatin volume. Observations of anticancer drugs’ distribution in cells/celluar nuclei are rare because such studies require an application of nanoscale analytical techniques. With infrared nanospectroscopy we have detected discrete foci of the platinum drug in single chromosomes (Figure 6) and isolated cellular nuclei (Supplementary Figure S14)

Transmission Electron Microscopy/nano Secondary Ions Mass Spectrometry (TEM/NanoSIMS) studies have confirmed an accumulation of concentrated Pt hotspots in nuclei of A2780 cells treated with cisplatin (88). Discrete foci in cellular nuclei have also been observed in other studies of cisplatin distribution in cancer cells (88–90). Discrete accumulation of platinum anticancer drugs (cisplatin and [{*cis-*Pt(NH_3_)_2_}_2_(μ-OH)(μ-tetrazolato-*N2*,*N3*)]^2+^ (5-H-Y) ) was observed also with scanning X-ray fluorescence microscopy (SXFM) (91). While TEM, nanoSIMS and SXFM correlated the distribution of Pt with phosphorus-rich chromatin regions, confirmed the binding affinity of the Pt-compounds DNA, and allowed for observation of Pt hotspots observation in cellular nuclei but they did not explain explicitly localised binding of Pt-drugs to DNA. Hou *et al.* based on AFM studies proposed a model, which explain DNA condensation caused by cisplatin (92). According to AFM observations cisplatin/oxaliplatin di-adducts induce local distortions of DNA and then through formation of distant crosslinks micro-loops of ∼20 nm appear. In the next step further crosslinks, lead to large aggregates and then a compact globule. Hou *et al.* postulated that this mechanism explains also the effect of other platinum anti-cancer drugs that are analogous to cisplatin (92) such as Pt-103. Our studies involved also an investigation into interaction between DNA pUC 19 plasmid with 200 mM of Pt-103 in order to confirm that the drug condenses DNA, leading to compact globule formation. Globular features were observed in AFM topographies of DNA incubated with Pt-103 for 2 and 12 h as presented in Supplementary Figure S15.

To determine the type of chromatin that preferentially binds to the Pt compound AFM-IR spectra were collected very close (at a distance <200 nm) to the places in which the drug was bonded. Around 100 spectra were collected from eight various chromosomes and modeled using PCA to assist in discriminating eu- from heterochromatin. The PCA model was constructed by combining the euchromatin and heterochromatin spectra used for generating the model in Figure 4 with the 50 spectra mentioned above (Figure 7). The first Principal component explains 87% of the total variance and is related to the differences between single chromosomes. Such differences include the size/thickness and density of the chromosome, which depends on the chromosomal stage (early/late metaphase) (3,4,11–13). However, the loadings plot shows that PC-2 (explaining 7% of total variance) and PC-3 (2%) are related to the spectral differences between eu- and heterochromatin. The model shows spectra collected from chromatin interacting with the drug clustering with the spectra of heterochromatin. PC-2 is negatively correlated to the CH_3_ band at 2952 cm^−1^ (heterochromatin) and positively correlated to a band at 2960 cm^−1^ (euchromatin) indicating two different chemical environments for the CH_3_ groups. This shift is significant in separating eu- and heterochromatin along PC2 in the PC1 versus PC2 scores plot. Similar loadings were observed in the previous PCA model applied to spectra collected from just one chromosome that distinguished euchromatin from heterochomatin (Figure 4D and E). The results indicate that Pt103 preferentially binds to heterochromatin and demonstrates the potential of AFM-IR to detect drug-binding sites in chromosomes. Heterochromatic regions tend to be rich with adenine and thymine pairs, therefore a detection of the Pt-103 mainly in heterochromatin areas confirmed the result of X-ray scattering studies (85), that adenine is the main target of this Pt compound attack. In contrast, cisplatin binds preferentially to guanine (93) and can be found rather in euchromatin areas (61), which are rich with cytosine and guanine pairs.

## CONCLUSION

In summary, the main scientific achievement of the work is the successful application of AFM-IR to detect DNA methylation status in single human metaphase chromosomes at a spatial resolution in the order of the AFM tip radius. This novel application of AFM-IR coupled with multivariate data analysis makes it possible to distinguish areas containing eu- and heterochromatin based on pure chemical information/the presence of the methyl motions, without any chemical labeling. Additionally, we have verified the ability of particular chromatin type to bind the anti-cancer drug Pt-103. This anti-cancer agent was found to bind preferentially to heterochromatin proving the versatility of our methodology and its usefulness to investigate the mechanism of action of chemotherapeutic compounds. The developed methodology can be used to routinely map eu- and heterochromatic areas in chromosomes and for the deep investigation into the interaction between a particular type of chromatin and chemotherapeutic drugs. The experimental approach presented here is an efficient tool for nucleic acid, genetic and also pharmaceutical research.

However, in order to explore fully chromatin structure and composition of a single metaphase chromosome, a systematic approach is required. This approach involves several important steps: AFM-IR mapping of methylene and phosphate motions, collection of at least 100 single spectra along a single chromosome, PCA analysis, and appropriate threshold choice in CH3/OPO map basing on PCA statistical classification of single spectra. Only following all these fundamental steps, this tool could be applied to detect sites of increased methylation and interaction of chemotherapeutic drugs in chromosomes. The extremely high spatial resolution of AFM-IR enables molecular imaging of a single metaphase chromosome, thereby opening up new possibilities for detecting chromosome abnormalities and drug interactions. At last, the development of AFM-IR working in a native liquid environment with vertical resolution of a few hundreds of nanometers has been recently demonstrated (41). The future development of resolution of AFM-IR in liquid promises new possibilities for detecting chromosome abnormalities and drug interactions simultaneously with conventional fluorescent methods.

## DATA AVAILABILITY

All data needed to evaluate the conclusions in the paper are present in the paper itself and the supplementary materials or available upon request from the authors.

## Supplementary Material

gkz630_Supplemental_File

## Notes

Present address: Ewelina Lipiec, Institute of Nuclear Physics, Polish Academy of Sciences, Krakow, PL-31342, Poland.
