# Supplementary material for: Infrared nanospectroscopic mapping of a single metaphase chromosome
Source: Nucleic Acids Res. 2019 Jul 25;47(18):e108. doi: 10.1093/nar/gkz630 (PMC6765102; doi:10.1093/nar/gkz630)
Supplement: gkz630_Supplemental_File [file gkz630_supplemental_file.pdf]

SUPPLEMENTARY DATA

Infrared nanospectroscopic mapping of a single metaphase chromosome

Ewelina Lipiec[1,2,3*], Francesco Simone Ruggeri[2,4], Carine Benadiba[2], Anna M. Borkowska[1], Jan D. Kobierski[4], Justyna Miszczyk[1], Bayden R. Wood[3], Glen B. Deacon[5], Andrzej Kulik[2], Giovanni Dietler[2] and Wojciech M. Kwiatek[1]

[1]Institute of Nuclear Physics, Polish Academy of Sciences, PL-31342 Krakow, Poland,

[2] Laboratory of Physics of Living Matter, Institute of Physics of Biological Systems, School of Basic Sciences, Ecole Polytechnique Federale de Lausanne, Lausanne, Switzerland,

[3]Centre for Biospectroscopy and School of Chemistry, Monash University, 3800, Victoria, Australia

[4]Department of Chemistry, University of Cambridge, CB21EW, United Kingdom.

[4]Department of Pharmaceutical Biophysics, Faculty of Pharmacy, Jagiellonian University, Medical College, Cracow, Poland

[5] School of Chemistry, Faculty of Science, Monash University, 3800, Victoria, Australia

CONTENT

S1Technical details of samples preparation and applied measurement procedures

S2 OPO laser power

S3 Resolution of AFM-IR

S4 AFM-IR band assignments for spectra of single chromosomes

S5 Second derivatives of AFM-IR spectra of metaphase chromosome (acquired from eu- and heterochromatin areas) and of methylated and unmethylated DNA

S6 Distribution of methylation in Xs female chromosomes

S7 Principal Component Analysis of metaphase chromosome and DNA spectra in fingerprint region

S8 Principal Component Analysis of methylated and unmethylated DNA spectra in fingerprint region

S9 Metaphase chromosomes fluorescence *in situ* hybridization (FISH) and Imaging

S10 Eu-end heterochromatin content in single metaphase chromosome

S11 Theoretical procedures in studies of interaction between chromatin and platinum anticancer drug Pt-103

S12 S 12 Pt-103 detection in single cellular nuclei

S13 Supplementary references

S1 Technical details of samples preparation and applied measurement procedures

Metaphase chromosomes isolation

Metaphase chromosomes were obtained from HeLa cells according to the following procedure. To enrich the number of cellular nuclei containing chromosomes in the metaphase stage, the cells were incubated with 5% colcemid for 4 hours. Xs human chromosomes derived from lymphocytes were obtained according to the same protocol, however colcemid was added to the medium 2 h before preparation. After drug treatment the cells were incubated with 0.75 mM KCl for 7 min and centrifuged (1). Then the cells were fixed in a mixture of acetic acid and methanol (3:1) and deposited on a cooled substrate – ZnSe crystal for AFM-IR measurements using nanoIR system and CaF2 windows (IR grade, Crystran) for measurements using nanoIR2 system. Chromosome from healthy female donors were investigated in accordance with the Human Bioethical Committee of the Regional Medical Board in Kraków (No. 124/KBL/OIL/2013).

Technical details of applied instrumentation and data acquisition

Both used lasers covered broad spectral ranges: OPO coupled to nanoIR2 uses two stages: 3600 cm^{-1} – 2234 cm^{-1} and 2000 cm^{-1} – 900 cm^{-1} and OPO coupled to nanoIR uses also two stages 3600 cm^{-1}–1610 cm^{-1}, 1610 cm^{-1} – 1000 cm^{-1}. The essential difference between those two systems lies in the geometrical arrangement of the IR illumination. In nanoIR system the sample is placed on a ZnSe prism and it is illuminated from the bottom, whereas the laser beam is reflected (total internal reflection) from the upper surface of the prism just below the sample, where an evanescent wave penetrates the sample-prism interface. Site illumination is applied in nanoIR2 system. Therefore this setup does not require usage of prisms and the chromosomes were fixed onto flat CaF2 windows for measurements with nanoIR2.

Experiments were carried out in contact mode using silicon (10 nm tip apex diameter, Res. Freq. 13±4 kHz, Spring. const. 0.07-0.4 N/m) and silicon gold coated PR-EX-nIR2-10 (20 nm tip apex diameter, Res. Freq. 13±4 kHz, Spring. const. 0.07-0.4 N/m) manufactured by Anasys instruments for nanoIR and nanoIR2, respectively.

The scan regions were related to chromosome size typically several microns, the scan rates were set between 0.02 Hz - 0.01 Hz.

The spectral resolution was ranging between ~4-8 cm^{-1} along the full spectra range of the laser (EKSPLA, Lithuania) (2).

Data post-processing

Single point spectra were smoothed using Savitzky-Golay algorithm (2nd order of polynomial, 3 smoothing points). PCA (cross-validation) was applied to not normalized spectra using Unscrambler 9.2 software (CAMO, Norway) on the smoothed data. AFM and infrared maps were analysed and processed using the SPIP software (Image Metrology, Denmark). The images were normalized to the applied laser power and the position of the images were correlated based on the topographies collected simultaneously with each absorption map. The z-offset for each map was changed in order to set minimum value to 1. Then the ratio (a map of IR absorption at 2952 cm^{-1} to a map of IR absorption at 1240 cm^{-1}) was calculated and nonlinear median filter was applied to 0.5 % of extreme values (the highest and lowest). The window of median filter was rectangular of size 15 pixels x 15 pixels, borders included.

PCA confirmed the chemical distinction in the banding pattern. The total scale CH_3/OPO ratio is in the range from 0 a.u. to 6 a.u. for thick (190–210 nm) chromosome 2, presented in Fig. 3. For thin chromosomes such as Xi, Xa and chromosome 3 (60 – 80 nm) the total scale CH_3/OPO ratio is in lower range of 0 a.u. – 2 a.u. The colour/intensity threshold was optimized for each chromosome separately because the IR signal is proportional to the thickness of the chromosome at each particular wavenumber but with a different angular coefficient. Thus, the absolute value of the ratio image is different depending on the chromosome thickness (Z direction in the AFM map). The thickness of each presented chromosome is given in the figure captions. Finally, the threshold was verified/improved based on results of multivariate data analysis described in the next part of the article. PCA classified eu- and heterochromatin spectra and the threshold on each map were chosen in order to demonstrate the areas of heterochromatin spectra location (in yellow colour) and the areas of euchromatin spectra location (in blue).

S2 OPO laser power

Averaged laser power from 24 measurements and its standard deviation are presented in the supplementary Fig. S1. Each AFM-IR spectrum was corrected to account for the varying laser power, but, it should be noted that during the measurement of such a small object like a chromosome (thickness less than 100 nm) only a very small amount of light is absorbed especially in the spectral regions where the laser power was relatively low. A drop in AFM-IR laser power in the 1230 cm^{-1} – 1190 cm^{-1} results in a significant difference between the spectra of chromosomes, DNA and cellular nuclei, which is addressed in Fig. S1. The laser power was measured

once per day, according to supplier's instructions, and the average laser power from 24 measurements and the standard deviation are also presented in the supplementary Fig. S1. Each AFM-IR spectrum was corrected to account for the varying power but it should be noted that during the measurement on such small objects like chromosomes (thickness usually less than 100 nm) only a very small amount of light is absorbed in the spectral regions where the laser power is relatively low. Interestingly, in each of three investigated independent chromosomes two types of spectra were collected (blue and red, Fig. 1).

For the spectral acquisition the laser power value was optimized for the entire fingerprint region 1800 cm^{-1}– 1000 cm^{-1}. Prior to AFM-IR mapping, the laser power value was optimized in order to increase both the signal-to-noise ratio and contrast between signal from the chromosome and background, for each independent wavenumber. Then, each acquired map was normalized to the laser power value. Therefore, the low laser power in the spectral range 1230 – 1190 cm^{-1} influences the spectral shape (peak ratio) but it does not affect the overall mapping of the chromosome. Additionally, the signal-to-noise ratio at 1240 cm^{-1} was much higher in comparison to the spectral range of O-P-O symmetric stretching region 1100–1080 cm^{-1}, as shown in Fig. 2.

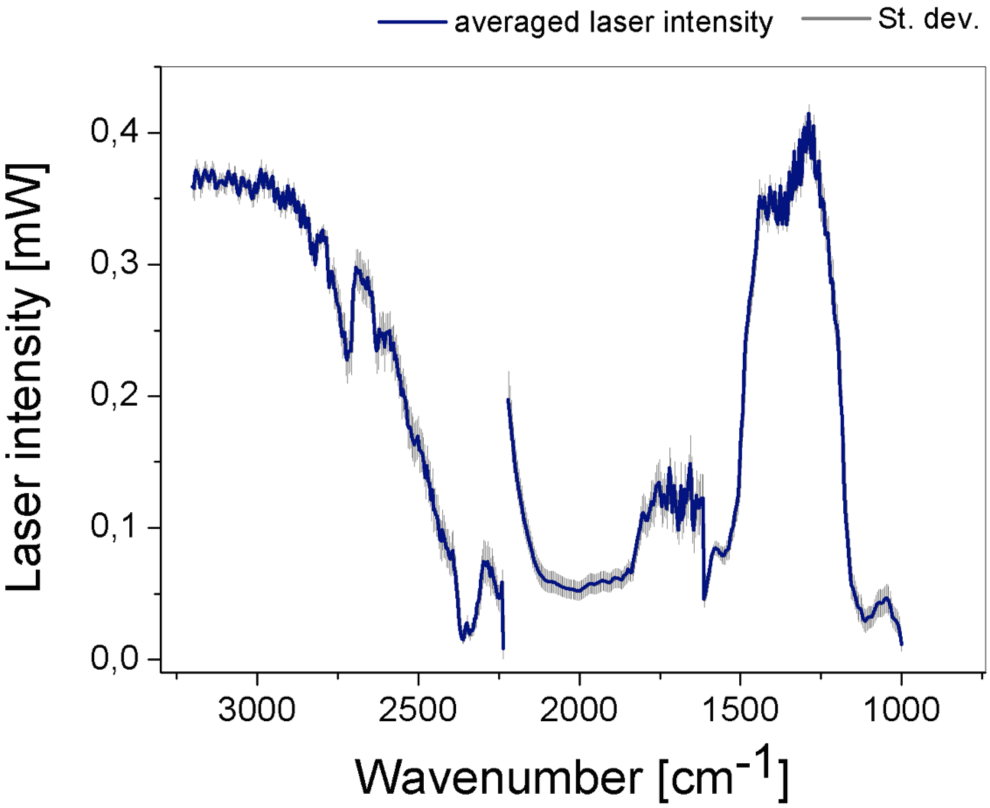

Supplementary Figure S1 Averaged relative laser power used during the collection of AFM-IR spectra.

S3 Resolution of AFM-IR

The spatial resolution on chromosomes

The AFM-IR technique is based on the photothermal induced resonance effect (PTIR) (3). If a pulse of IR light at a fixed wavenumber is absorbed by a sample, the local rise in temperature leads to a local photo-thermal expansion. The AFM tip enables the detection of this temporary dilatation of the scanned region with a lateral resolution defined in principle by the dimensions of the AFM tip at the nanometer scale and it allows reconstructing, together with the acquisition of conventional morphology imaging, the IR-absorption map of the sample. It has been demonstrated in the case of several biological samples that the resolution of the instrument is routinely in the order of 10-20 nm, which is indeed the typical radius of a conventional AFM tip (3–5).

In order to calculate how precisely we were able to detect the edges between hetero- and euchromatin areas, we applied a knife-edge method to our measurements. This methodology was applied to the image ratio at the wavenumbers corresponding to the IR absorption of CH_3

and O-P-O chemical bonds. Indeed, the analysis of the distribution of the CH_3/O-P-O absorption ratio provides pure chemical information about the methylation along the chromosome. Recently biochemical heterogeneity of mammalian cells was studied by Kennedy *et al*. Researchers applied infrared nanospectroscopy combined with topographic data. Their studies indicated that the changes in the cantilever deflection observed while scanning complex biological systems are not only the function of the material absorption coefficient but also the amount of material located under the tip. Furthermore, the normalization to the intensity of the O-P-O band (density of nucleic acids) allows avoiding the influence of sample thickness on the total intensity of the AFM-IR signal from the CH_3 groups.

In Supplementary Figure S2, one can see the AFM-IR map (CH_3/O-P-O ratio) of the chromosome and two intensity profiles extracted along the chromosome and along the substrate nearby (background). A first estimation of the spatial resolution was possible by measuring the minimal distance between the minimum and maximum signal at sharp borders between methylated and un-methylated types of chromatin. As it is possible to observe in the bottom of Fig. S2, where zoomed sections are shown, the spatial resolution is in the order of 12-25 nm. In order to estimate quantitatively and more accurately the spatial resolution, we performed a first derivative of each section, we fitted it by a Gaussian function and we measured its full width at half maximum (FWHM). This quantity is intimately related to the sharpness of the transition and to our spatial resolution, which was in the best case 12 ± 7 nm. The experimental error in determining the spatial resolution has been determined by considering the sum of the statistical error in determining the FWHM (≈1 nm), plus the sensitivity error due to the image pixelization (≈6 nm). In particular, in our specific case, it is not the tip size, but rather the pixel size in the maps that determines the ultimate spatial resolution measureable. Indeed, as stated by the Shannon-Nyquist theorem, the highest resolution that can be obtained in a raster map is twice the pixel size and it is at the best ≈12 nm in our maps (6). Finally, for the sake of rigor, we should specify that the knife-edge method is not applied to the raw data (AFM-IR signal), but to a divided/normalised image. For this reason, though we are still measuring accurately the spatial resolution, a further error in the order of 10 nm has to be considered in the experiment of the absolute position measurement in the map of the borders between methylated and un-methylated types of chromatin. Thus, the above discussion leads to an average ultimate spatial resolution in in our measurements of 15 ± 17 nm.

The intensity CH_3/OPO ratio in the substrate area was approximately 5 times lower than the signal collected from the chromosome (Fig. S2). This signal is a convolution of instrumental noise and low absorption of IR light in-cellular debris, which cannot be avoided during the sample preparation procedure.

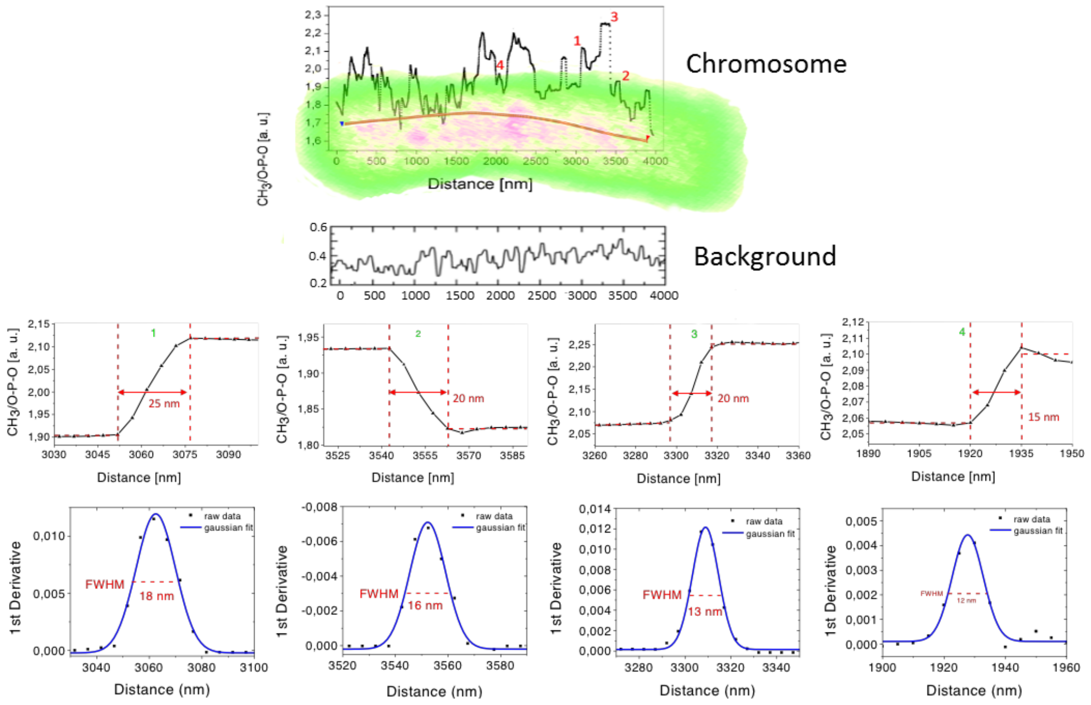

Supplementary Figure S2 The CH_3/O-P-O profile extracted along the metaphase chromosome and along background (substrate) and zoomed four exemplary slopes fitted with error complement function.

In order to explore contrast between eu- and heterochromatin zoomed CH_3/O-P-O map of the flat area of the chromosome (Fig. S3) was analyzed. Map was acquired with bottom illumination set-up (nanoIR). Single raw (not normalized) spectra collected from eu- and heterochromatin areas are presented in Fig. S3a. Spectral differences described for averaged spectra (Fig. 3) in the manuscript such as the intensity of methyl and methylene stretching region at 2927 cm^{-1} and 2960 cm^{-1} - 2952 cm^{-1} and spectral shape of the methyl deformation mode from cytosine at 1408 cm^{-1} can be observed here as well. The shift of the CH_3 asym. str. position from 2960 cm^{-2} (euchromatin) to 2952 cm^{-2}, explored by PCA is also visible in single spectra presented in Fig. S3a. The CH_3/O-P-O section extracted along flat area of the chromosome and two slopes (first derivative of the section) fitted with Gaussian function are shown in Fig. S3c. Calculated FWHM values are comparable to these obtained in previous paragraph.

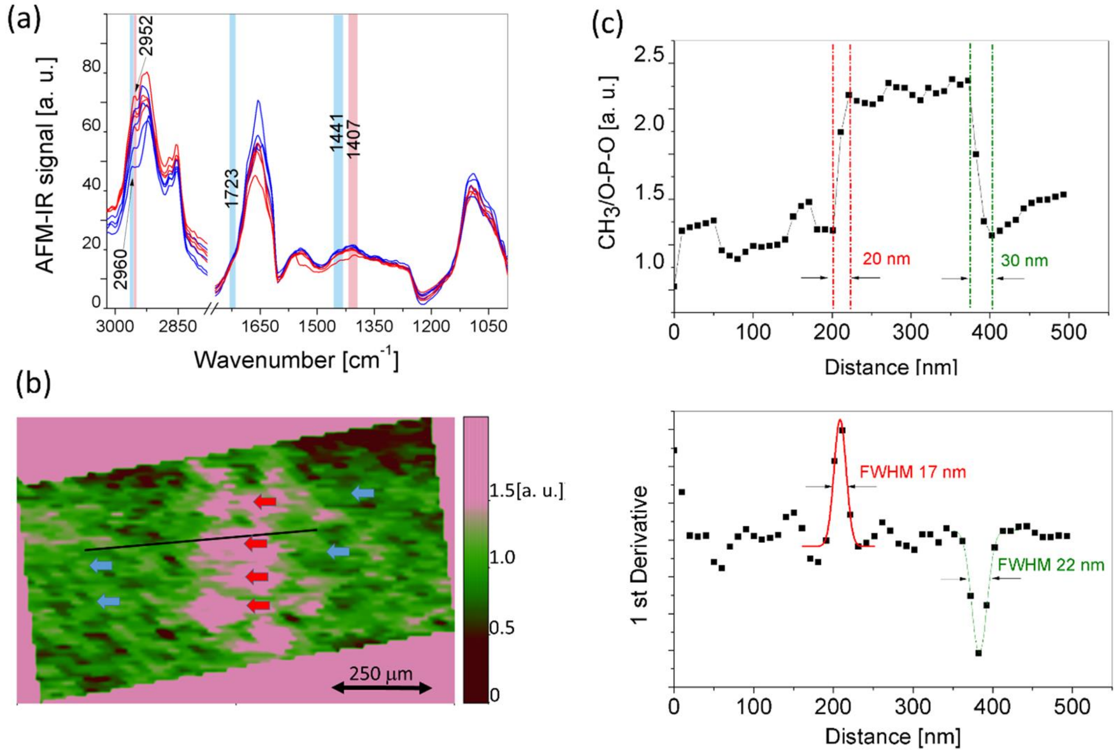

Supplementary Figure S3 AFM-IR resolution: (a) raw spectra acquired from eu- (blue) and heterochromatin (red), (b) zoomed area of CH_3/O-P-O map with marked places of single spectra collection and profile extraction, (c) The CH_3/O-P-O profile extracted along flat area of the chromosome and zoomed two slopes fitted with error complement function.

Chromosome thickness (100 – 250 nm), is one of the main factors, which determines the resolution of eu- heterochromatic boundaries visible in CH_3/O-P-O distribution maps. Therefore in order to demonstrate the accuracy of our methodology we resolved eu- heterochromatic areas in small (~ 60 nm height) chromosome fragments called double minutes. Double minutes are one of several types of chromosomal aberrations. This data was collected with top illumination set-up (nanoIR2). Fig. S4 show the results. The CH_3/O-P-O profile extracted along eu- heterochromatic areas of minutes (Fig. S4e) demonstrates the CH_3/O-P-O intensity difference between these two types of chromatin. The boundary is not as sharp as in whole chromosomes. FWHM here is equal to 37 ± 12 nm. Lower SNR than in chromosome maps is responsible for a blur of the border between eu- and heterochromatin in CH_3/O-P-O intensity map of double minutes.

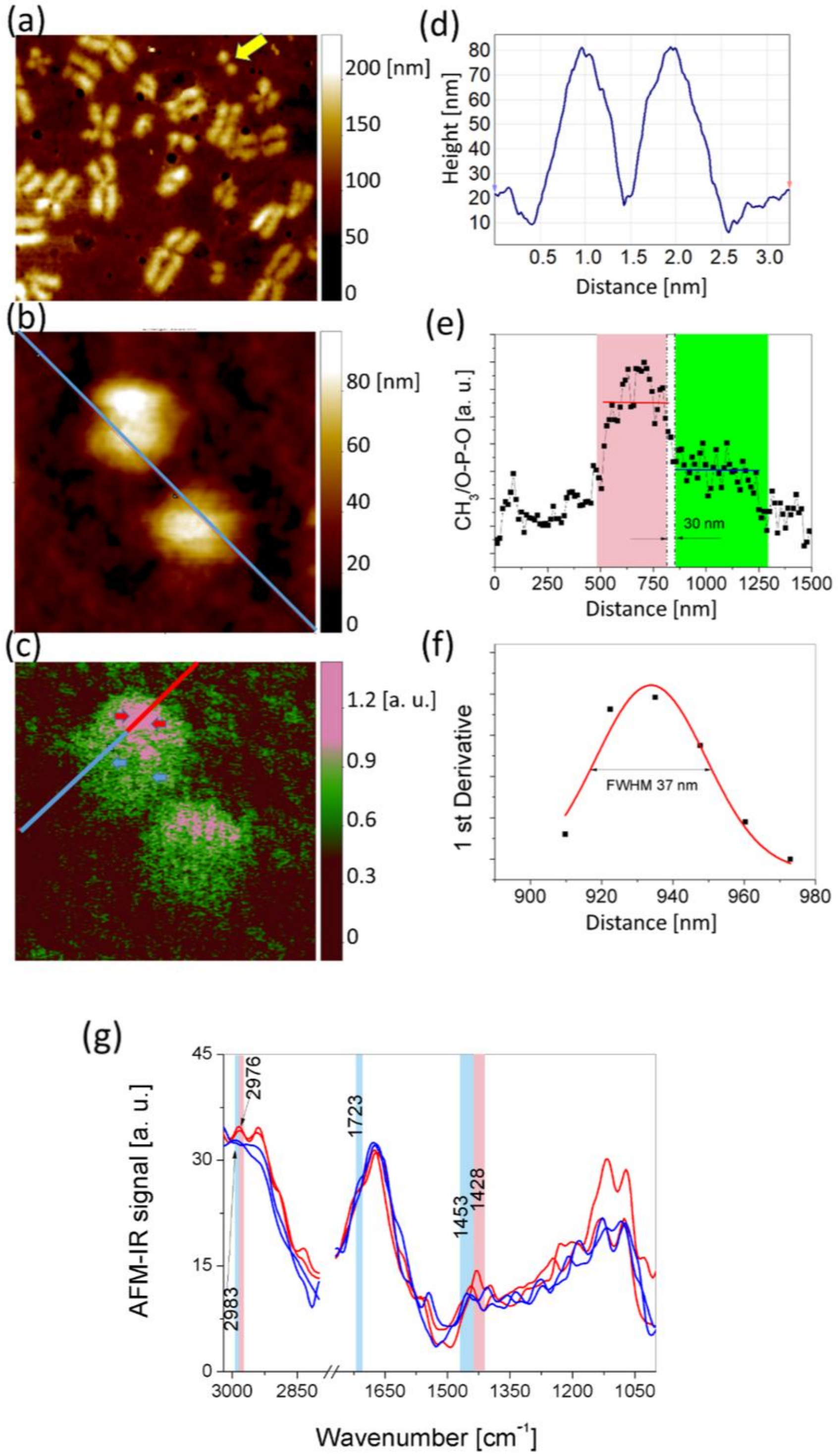

Supplementary Figure S4 AFM-IR mapping of double minutes: (a) AFM topography of metaphase fragment; minutes are highlighted by yellow arrow, (b) AFM topography of minutes, (c) CH_3/O-P-O map of minutes, (d) cross-section along minutes, (e) The CH_3/O-P-O profile extracted along minutes, (f) Gaussian function fitted to the zoomed slope between eu-and heterochromatic areas of minutes; profiles extraction lines are marked in b and c, (g) AFM-IR spectra collected from places highlighted by arrows.

The spatial resolution on fibrillar samples

In order to illustrate the ultimate spatial resolution of AFM-IR, we report here what we demonstrated in our previous studies on the chemical characterisation of individual amyloid fibrils (7), where an AFM-IR system with top illumination and an AFM tip with a nominal diameter of 30 nm was used (PR-EX-nIR2, Anasys, USA). Spatial resolution of an optical image can be also defined as the closest distance at which two different objects can be still distinguished. In the Fig. S5 a-b, we report a 3-D morphology and IR map of a bundle of amyloid fibrils. In particular, the image shows closely spaced fibrils. Extracting the cross-sectional profile of height (blue) and IR absorption (red) at the same position shows that the morphology and infrared signal are well correlated, furthermore two fibrils at a distance of approximately 10-30 nm can be fully distinguished. In conclusion, as previously demonstrated (7–11), tough the laser spot has a diameter of in the order of tens of micrometres, AFM-IR exploits the tip of the AFM as sensor of IR absorption and enable to acquire chemical information with the ultimate resolution being defined the sized of the AFM tip.

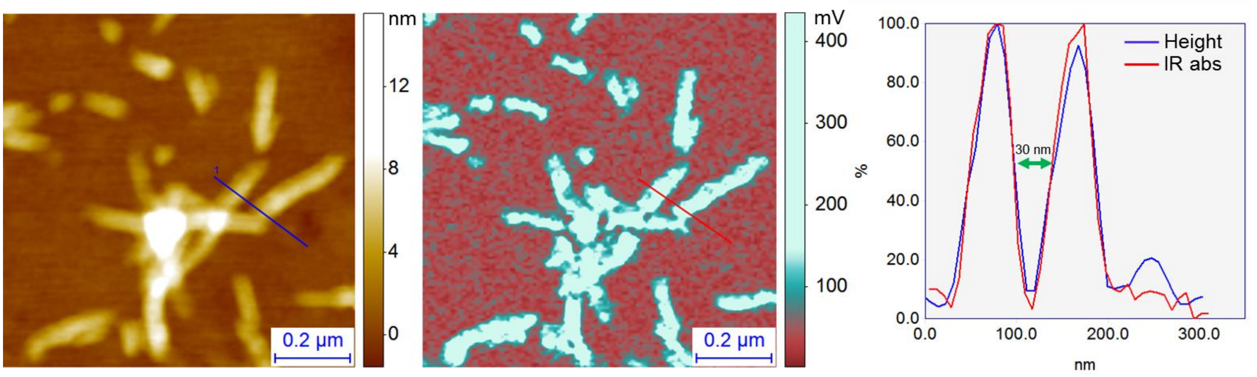

Supplementary Figure S5 AFM-IR mapping of amyloid fibrils. A) AFM morphology map, B) IR absorption map in the C=O stretching (1630 nm-1). C) Correlation between the height and IR absorption signal of two closely spaced fibrils.

S4 AFM-IR band assignments for spectra of single chromosomes

Table S1 AFM-IR band assignments for spectra of single chromosomes, cellular nuclei, nucleic acids and their components.

	Wavenumber [cm $^{-1}$]	Assignment
1	2960, 2952	$v_{asym}(CH_3)$(12–14)
2	2927	$v_{asym}(CH_2)$(12–14)
3	2879	$v_{sym}(CH_3)$(12–14)
4	2850	v_{sym} (CH_2)(12–14)
6	1715	v(C=O) nucleic acids: G, T, C, base–stacking mode sensitive to DNA conformation(12–14)
7	1695–1610	Histones: Amide I (predominantly v(C=O)), sensitive to hydration (15–17)<br>1690 antiparallel β–sheet from from proteins (15–17)<br>1670 β–turns and bends (15–17)<br>1655 α–helical structure (15–17)<br>1640 random coil (15–17)<br>1630 native parallel β–sheet structure (15, 16)<br>DNA: v(C=O), $\delta_r(NH_2)$ dT, dG, dC, C/G/T, v(C=C), δ_s (NH_2) (14, 18, 19)
	1570	A/C/G/T rings (18, 19)
8	1536	Amide II predominantly δ(N–H) coupled to v(C–N) sensitive to hydration<br>in–plane ring str. dC, T (18, 19)
9	1470	$\delta(CH_2)$ sensitive to hydration (20)
10	1440	right-handed helices (15, 20)
11	1408	$\delta(CH_2)$ α–methylene, C2' /C3' -endo deoxyribose (15, 20)
12	1300	Amide III, in phase combination of δ_s(N–H) and v(C–N) and $\delta(CH_2)$ sensitive to secondary structure folding (13, 17)
13	1230-1240	v_{asym} (O–P–O) DNA backbone, sensitive to conformational changes (21, 22)
14	1172	v_{asym} (O–P–O) A form RNA, C3' endo-sugar phosphate from DNA (14)
15	1125	v(P–O–C), v(O–C), v(C–C–O–C) ribose–DNA backbone (12, 14)
16	1080-190	v_{sym} (O–P–O) DNA backbone, sensitive to conformational changes (21, 22)
17	1060	v(C–O) DNA backbone (21, 22)

v – stretching, v_{sym} – symmetric stretching, v_{asym} – asymmetric stretching, δ – bending, δ_s– scissoring (in–plane bending), δ_r – rocking (out–of–plane bending)

S5 Second derivatives of AFM-IR spectra of metaphase chromosome (acquired from eu- and heterochromatin areas) and of methylated and unmethylated DNA

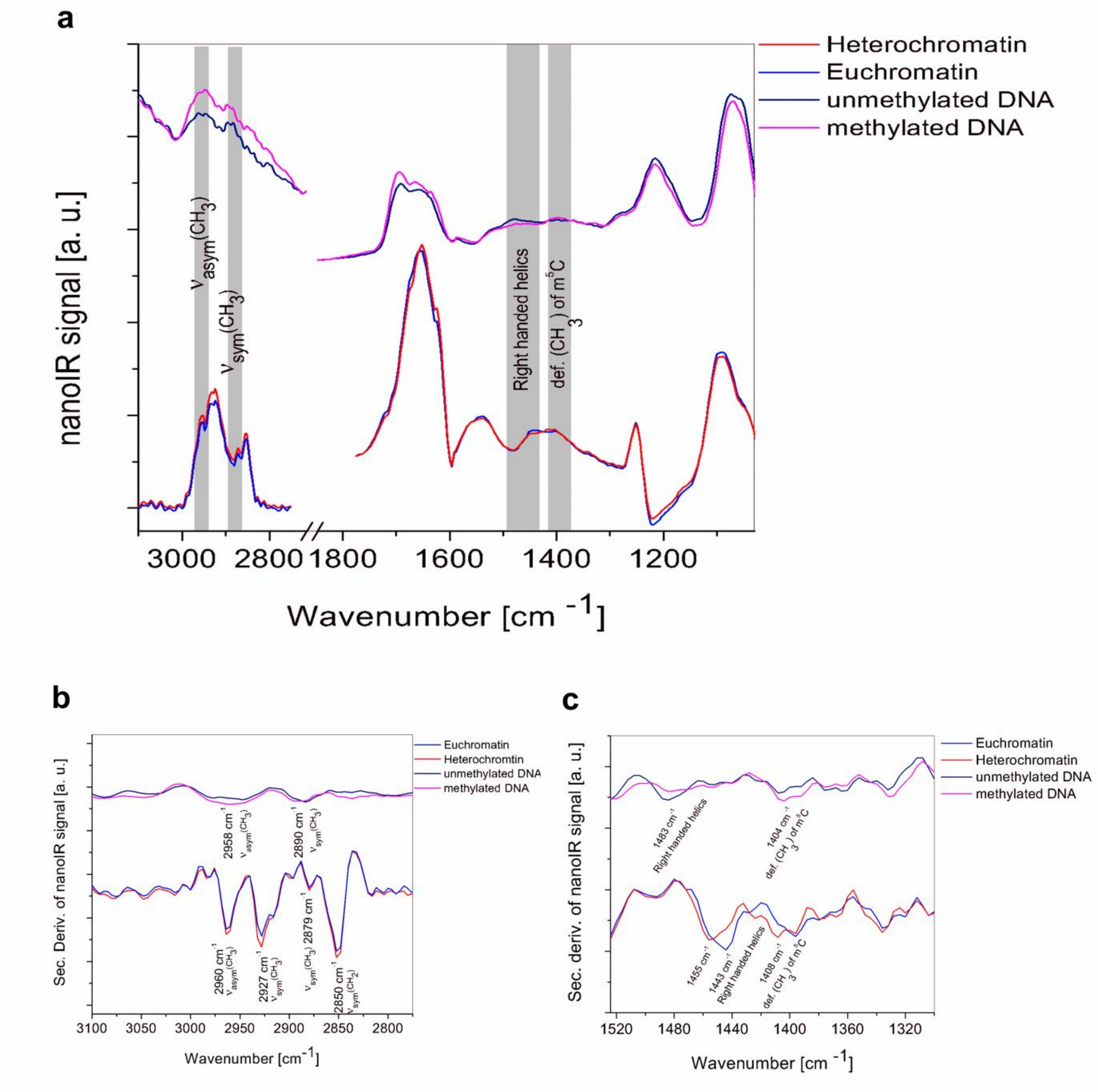

Supplementary Figure S6 A comparison of AFM-IR infrared spectra collected from areas containing euchromatin, heterochromatin, methylated DNA and unmethylated DNA, (a) 80 averaged spectra collected from heterochromatin, 80 averaged spectra collected from euchromatin, 10 averaged spectra collected from methylated DNA and 10 averaged spectra collected from unmethylated DNA, (b) and (c) second-derivatives of spectra presented in a.

S6 Distribution of methylation in Xs female chromosomes

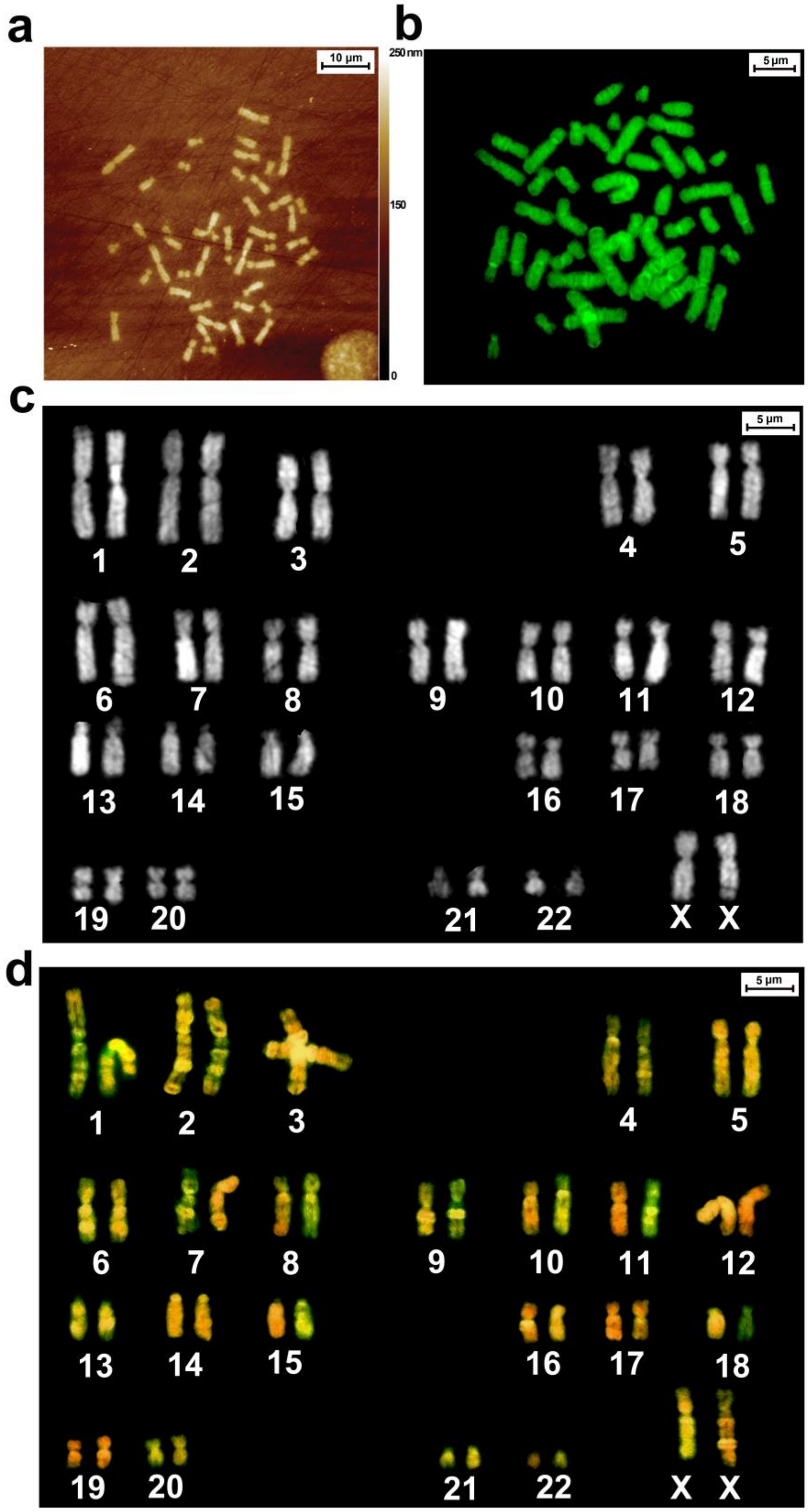

Supplementary Figure S7 Human metaphase chromosomes from a female patient from lymphocytes: (a) AFM topography, (b) fluorescent image of chromosomes stained using anti-5-methylocytosine antibody, (c) a karyogram of chromosomes based on the AFM image a, (d) karyogram of chromosomes based on the fluorescent image b.

To confirm achieved results by AFM- IR technique, chromosomes isolated from an anonymous patient were directly immunofluorescently stained using the Imprint® Monoclonal Anti-5-methylcytosine (33D3) antibody produced in a mouse (Sigma- Aldrich, SAB4800001) and counterstained using propidium iodide (Sigma- Aldrich) to better visualize bands and distribution of methylation of DNA (Supplementary Fig. S7b). The first step of staining was incubation in saline-sodium citrate (SSC) for 10 min in 37°C. The sample was then incubated in pepsin solution (20 μg/ml in 0.1N HCl, Sigma- Aldrich) also in 37°C. After incubation for 10 min, sample was washed using phosphate buffered saline (PBS) (Sigma- Aldrich) and dehydrated using following concentrations of ethanol: 50%, 70%, 100%. Subsequently, the sample was rehydrated in PBS and incubated with anti- 5mC antibody (92 μg/ml) and propidium iodide (0.25 μg/ml) for 3h in 37°C. Photos were taken using the Metafer system (MetaSystems) with a fluorescent microscopic module (63x) and manual MMC module using ISIS software. Based on the fluorescent photos (63x) obtained in different channels for fluorescein (FITC) and propidium iodide stain, a karyogram of chromosomes was set using the same method as before (Supplementary Fig. 7 d). Two X chromosomes were also typed based on a karyogram (Fig. 7 c,f) and the distribution of methylation based on fluorescence staining was compared with the distribution based on the AFM- IR spectroscopic data (Fig. 7b-c, e-f).

S7 Principal Component Analysis of metaphase chromosome spectra in the fingerprint region

Principal Component Analysis was applied in the second spectral range from 1750 cm^{-1} to 1230 cm^{-1}. The PCA results generated within this spectral range are presented in Supplementary Fig. S8. The Scores Plot shows two clusters of spectra: i) collected from euchromatin and ii) from heterochromatin. Each PC is dominated by spectral features related to the Amide I band (from histones) in the spectral range from 1700 cm^{-1} to 1600 cm^{-1}. PC-1 and PC-3 (6 % of total variance) indicate that bending from the methyl group from methylocytosine at 1407 cm^{-1} is also responsible for the clustering in the scores plot. This band position and intensity is related to DNA conformation and DNA packing (23) in chromosome structure.

Based on the PCA analysis of the chromosome spectra in the fingerprint region, it was possible to observe chemical modifications in histone structure in eu- and heterochromatin. Intensity changes of C=O at 1723 cm^{-1} also influenced the clustering of the spectra. This spectral change could be related with acetylation of histones in euchromatin (24–26). PC-1 (61 % of total variance) and PC-3 (6 % of total variance) indicate that bending from α–methylene group in cytosine at 1407 cm^{-1} (15, 20, 27) is also responsible for the clustering in the scores plot (Fig. S8)

indicting different sugar-base conformations (20) related to the degree of DNA packing in eu- and heterochromatin. PCA performed on DNA spectra in the fingerprint spectral range indicated a different sugar-base conformation associated with the spectral change observed at 1408 cm^{-1} (20), which difference is a significant factor affecting the clustering of spectra collected from methylated and un-methylated DNA. This provides a unique insight into chromatin structure at the molecular level that is only achievable using AFM-IR spectroscopic methods.

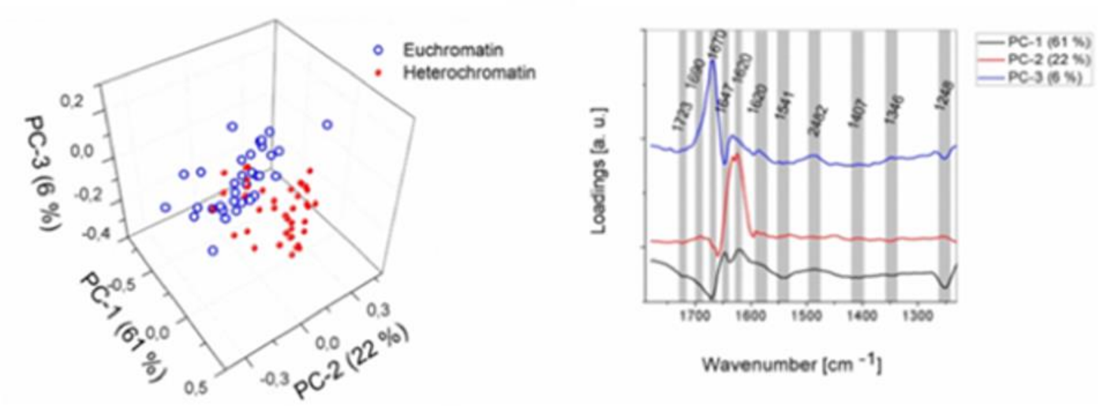

Supplementary Figure S8 Results of the PCA performed on spectra collected from a single chromosome in spectral range from 1750 cm^{-1} to 1230 cm^{-1}, (a) scores plot of spectra collected from a single chromosome,(b) Loadings Plot corresponding to a.

S8 Principal Component Analysis of methylated and un-methylated DNA spectra in the fingerprint region

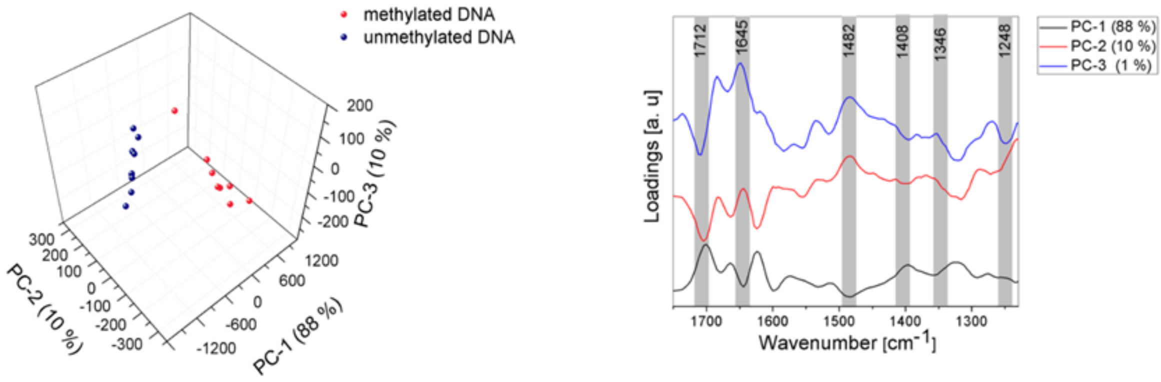

Supplementary Figure S9 The results of the PCA performed on spectra collected from dried droplet of methylated and unmethylated DNA (a) scores plot (b) loadings plot corresponding to a.

In the fingerprint region each PC indicates that the base stacking mode at 1712 cm^{-1} is responsible for the clustering observed in the scores plot (Supplementary Fig. S9a). Intensity changes in this mode are considered to be related to different levels of DNA packing (28)**.** Therefore, this result confirms the hypothesis that AFM-IR spectroscopy can detect lightly packed DNA (typical for euchromatin) from tightly packed DNA (heterochromatin). In addition, all PCs indicate intensity changes in the methyl deformation band of cytosine at 1407 cm^{-1} and 1346 cm^{-1} (15, 16, 27, 29)**,** which are also related to the DNA structure and packing, and intensity changes of the band assigned to right handed helices at 1440 cm^{-1} (27, 29).

S9 Metaphase chromosomes Fluorescence in situ hybridization (FISH) and Imaging

FISH was performed using the commercially available Red Alu probe (Chrombios GmbH). The repartition of the Alu sequences along the chromosomes corresponds to GC rich areas, and provides a R-band profile linked to euchromatin repartition (30). Briefly, 4 µL of Alu Red probe was added to a slide containing human chromosomes and a 16 mm coverslip was placed on the sample and sealed with nail polish. Denaturation of the DNA was performed at 72°C for 5 minutes. Then, the slide was incubated at 37°C overnight to promote the hybridization process. The coverslip was carefully removed from the slide and the slide was placed into 2X SSC buffer bath for 5 min at RT. The slide was transferred into a bath of 0.4X SSC /0.1% Tween pre-warmed solution at 70°C for 1 min to remove un-hybridized probes. The slide was rinsed in a bath with 2X SSC for 5 min at RT before counterstaining with Dapi (Sigma-Aldrich) at 0.5 µg/mL for 15 min. Finally, the slide was rinsed with 2X SSC and mounted using Mowiol solution.

The application of Alu-DNA as the probe, produces a pattern of hybridization signals similar to negative Giemsa banding (31, 32). The Alu probe (red) was used in FISH staining, to highlight the euchromatin areas of chromosomes and fluorescent dye. DAPI (blue) was used as a counterstain but this approach did not achieve the same spatial resolution as AFM-IR, since lateral resolution of fluorescent staining is diffraction limited. Moreover, the FISH technique is reliant on the detection of cytosine-guanine pairs as opposed to AFM-IR that relies on the detection of the degree of methylation. The latter is more important in discerning the active (euchromatin) from non-active (heterochomatin) DNA. Euchromatin is characterized by trimethylation at H3K4, H3K36 and H3K79 and heterochromatin is enriched in trimethylation at H3K9, K3K27, and H4K20.(33)

Imaging was performed using an inverted Zeiss Axiovert Z1 system with a CoolSnap camera and a 63x, oil immersion objective (N.A. 1.4).

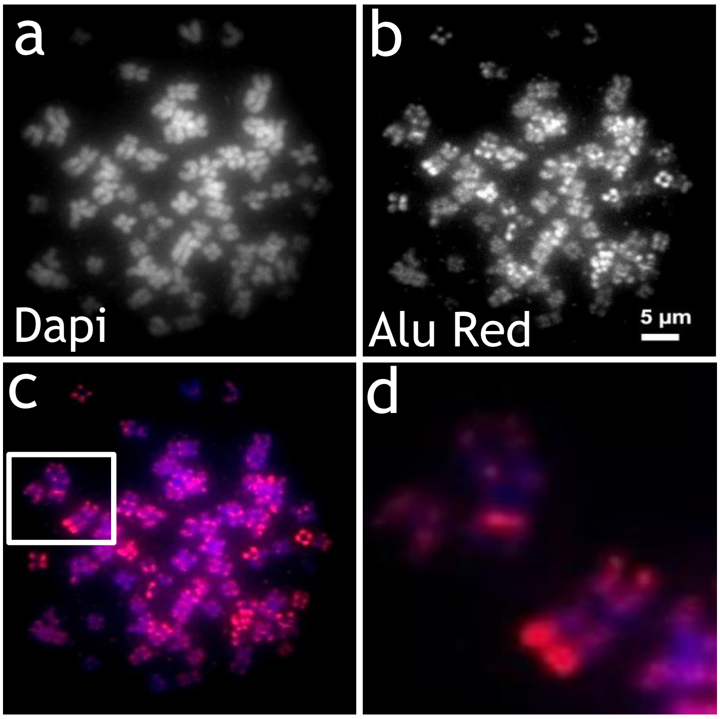

Supplementary Figure S10 FISH with repetitive Alu probes on human chromosomes. (a) DAPI counterstain on human chromosomes, (b) FISH using the dispersive repetitive Alu probe providing R banding pattern in red, (c) Merge of DAPI/Red-Alu probe on chromosomes. The distribution of Alu probe (red color) corresponds to GC rich areas, and provides a R-band profile linked to euchromatin repartition, (d) High magnification of the area defined in c panel. The scale bar represents 5μm.

S10 Eu and heterochromatin content in single metaphase chromosome

In order to estimate content of heterochromatin in measured chromosomes, using AFM- IR spectroscopy, AFM- IR maps were extracted and analyzed using *ImageJ* software (*NIH*, open source, Fig. 11 a-e). First, background of maps was cut, image color type was changed for 8-bits. The threshold of color intensity was chosen (in a range of 0- 220) and the area of each chromosome was calculated using *Analyze Particles* option. Following, in the AFM- IR map, using *TresholdColour Plugin*, yellow color was chosen (characteristic for heterochromatin) and then the area of image fragment occupied by this color was calculated also using *Analyze Particles* option. Finally, the ratio of the area of image fragment occupied by yellow color and the area of the whole chromosome was calculated and an estimation of heterochromatin content

was obtained (Table S2, Fig. S11 a-e). The obtained results were compared with values given by International Human Genome Sequencing Consortium of heterochromatin and euchromatin content in human chromosomes (presented graphically in Fig. S11 f and in Table S2) (34–36). As it could be seen in the Table S2, obtained by the AFM- IR method values are slightly larger than values obtained by the International Human Genome Sequencing Consortium. A difference which can be caused by the fact that in AFM- IR technique both methylation of DNA and histones is followed. Additionally, as explained in the main text of the manuscript observed difference is related to that compared chromosomes were extracted from different cells (different donor, growth phase and metabolic activity etc,). What is more, it can be seen that content of eu- and heterochromatin is different in different human chromosomes and additionally the ratio of heterochromatin to chromatin content is not constant in all chromosomes (Fig. S11 f). This phenomenon can be also observed when AFM- IR nanospectroscopy is applied to identify heterochromatin distribution on human chromosomes. It manifests itself in a different scale of the images when 2952/1240 ratio is calculated (Fig. 3, 4, 5).

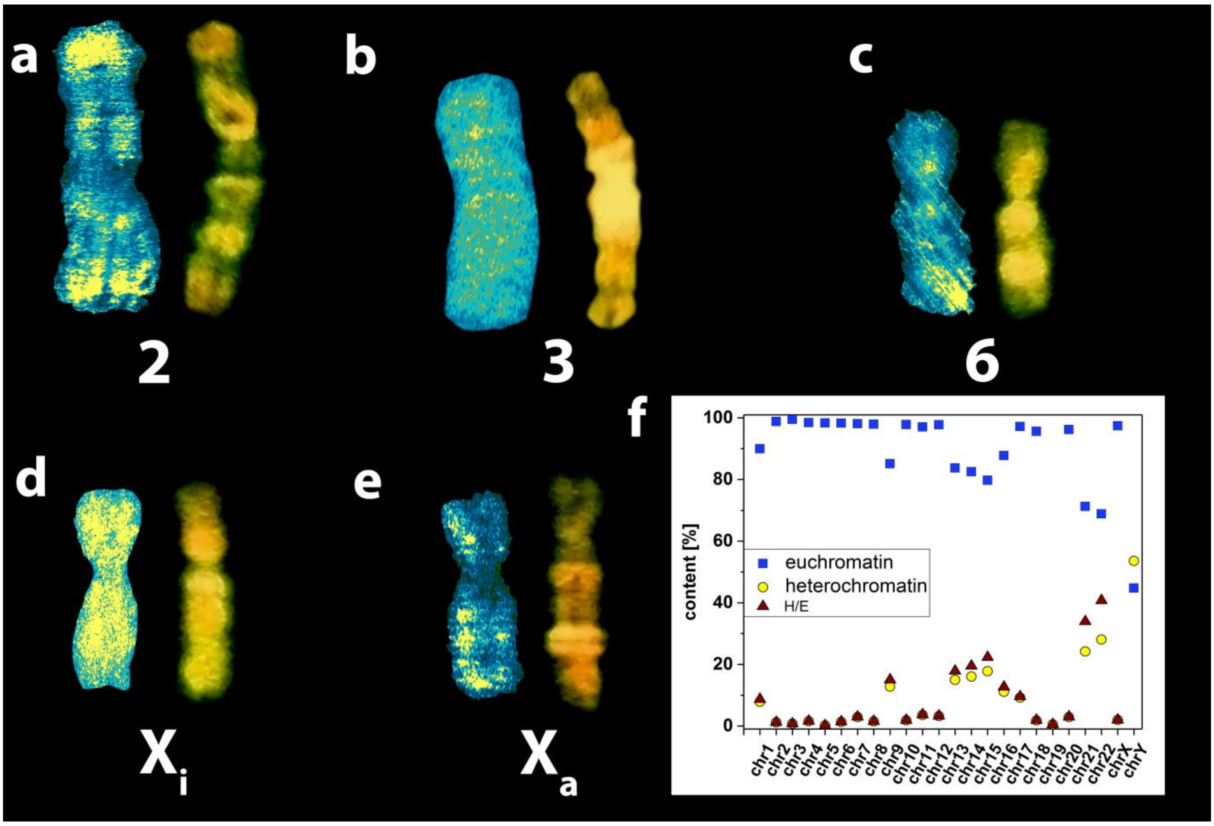

Supplementary Figure S11 An estimation of the content of euchromatin and heterochromatin in chosen human chromosomes based on AFM-IR maps (CH_3/OPO ratio left). Fluorescent staining (5mC right) was added in order to compare the banding pattern; **a** chromosome no 2,

b chromosome no 3, **c** chromosome no 6,**d** X_i inactive X chromosome, **e** X_a active X chromosome, Content of eu- and hetrochromatin and their ratio calculated for each chromosome based on literature.(34–36)

Table S2 An estimation of the content of euchromatin and heterochromatin in chosen human chromosomes

**Chromosome**	**Heterochromatin content [%]**	
	**Literature data** (34–36)	**AFM- IR maps (2952/1240 ratio)**
**2**	1,19%	2,13%
**3**	0,76%	2,14%
**6**	1,35%	1,88%
**Xi**	-	52,15%
**Xa**	1,94%	3,30%

S11 Theoretical procedures

In the present work, calculations in liquid phase were carried out using the M062X method (37) with cc-pVDZ basis set (38), for both geometry optimizations and frequency calculations. For the Pt atom the LanL2DZ effective core potential was used. All these quantum chemical calculations have been conducted using the GAUSSIAN09 software package (39). Raw frequencies values have been scaled by the factor 0.955 [http://cccbdb.nist.gov/vibscalejust.asp] to eliminate known systematic errors resulting from incomplete incorporation of electron correlation and the use of finite basis sets.

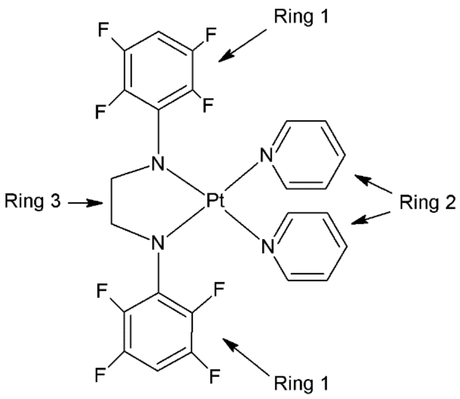

Supplementary Figure S 12 Structural formula of the Pt-103.

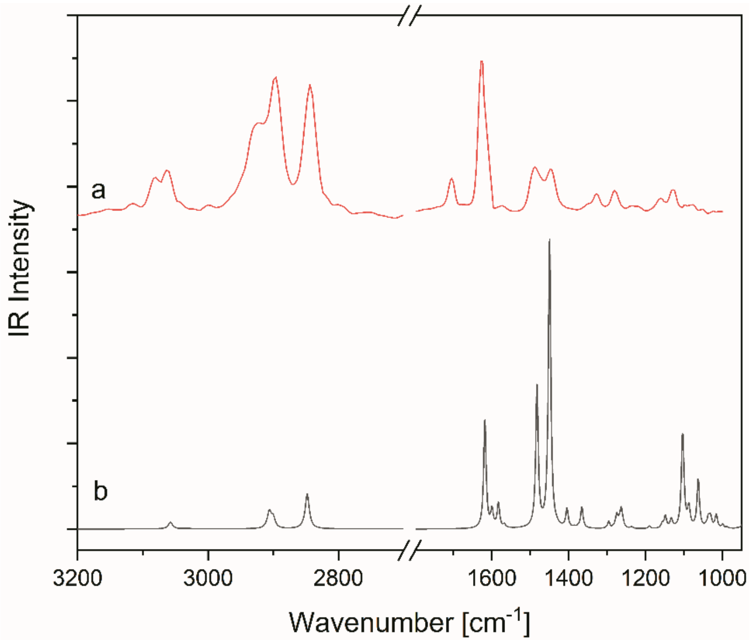

Supplementary Figure S 13 (a) Experimental AFM-IR spectrum collected from pure Pt103, theoretical spectrum of Pt-103 compound presented in S9.1,

Supplementary Table S3 A comparison of theoretical and experimental vibrational modes of Pt-103 compounds (from Fig. S12) with the assignment: ν_s – symmetric stretching, ν_{as} – asymmetric stretching, α – scissoring, ρ – rocking, τ – twisting

Vibrational Wavenumber (cm^{-1})		Assignment
Experimental	Theoretical	
3062	3058	ν(C-H) in ring 1
	3055	ν(C-H) in ring 2
2924	–	–
2898	2903	ν_{as}(C-H) in ring 3
2840	2848	ν_s(C-H) in ring 3
1704	–	–
1628	1618	ring 1 breathing
	1599	ring 2 breathing
1576	1583	ν_{as}(C-C) in ring 1
1488	1482	ν(C-C), ν(C-F) in ring 1

1446	1449	ν_s(C-C), ν(C-N) in ring 1,<br>ρ(C-H) in ring 3,<br>ρ(Pt-N)
	1431	ν(C-C) in ring 2,<br>ρ(Pt-N)
	1404	ν(C-C) in ring 2
1328	1376	α (C-H) in ring 3
1280	1263	ρ(C-H) in ring 3
1232	1236	ν(C-C) in ring 2
1132	1103	ν(C-F) in ring 1,<br>τ(C-H) in ring 3,<br>ρ(Pt-N)

S12 Pt-103 detection in single cellular nuclei

We have applied infrared nanospectroscopy in order to demonstrate the drug distribution in isolated cellular nuclei at the nanoscale. HeLa cells were incubated with 50 μM of Pt-103 for 4 hours and then cellular nuclei isolation was performed according to the procedure previously described (40, 41).The distribution of infrared absorption at 2916 cm^{-1} as a marker of the Pt-103 compound was mapped in zoomed areas of nuclei. Exemplary results are presented in Fig. S13. An accumulation of Pt-103 compound in nucleolus and localized in discrete foci Pt-103 accumulation in nucleus volume were detected. Spectra collected from these foci in comparison with spectra acquired at neighboring areas and AFM-IR spectrum of pure Pt-103 are also presented in Fig. S13. In spectra acquired from places of discrete accumulation of Pt- compound spectral features characteristic for spectrum of pure Pt-103 including 1132 cm^{-1}, 1278 cm^{-1}, 1628 cm^{-1}, 2840 cm^{-1} and 2916 cm^{-1} can be observed.

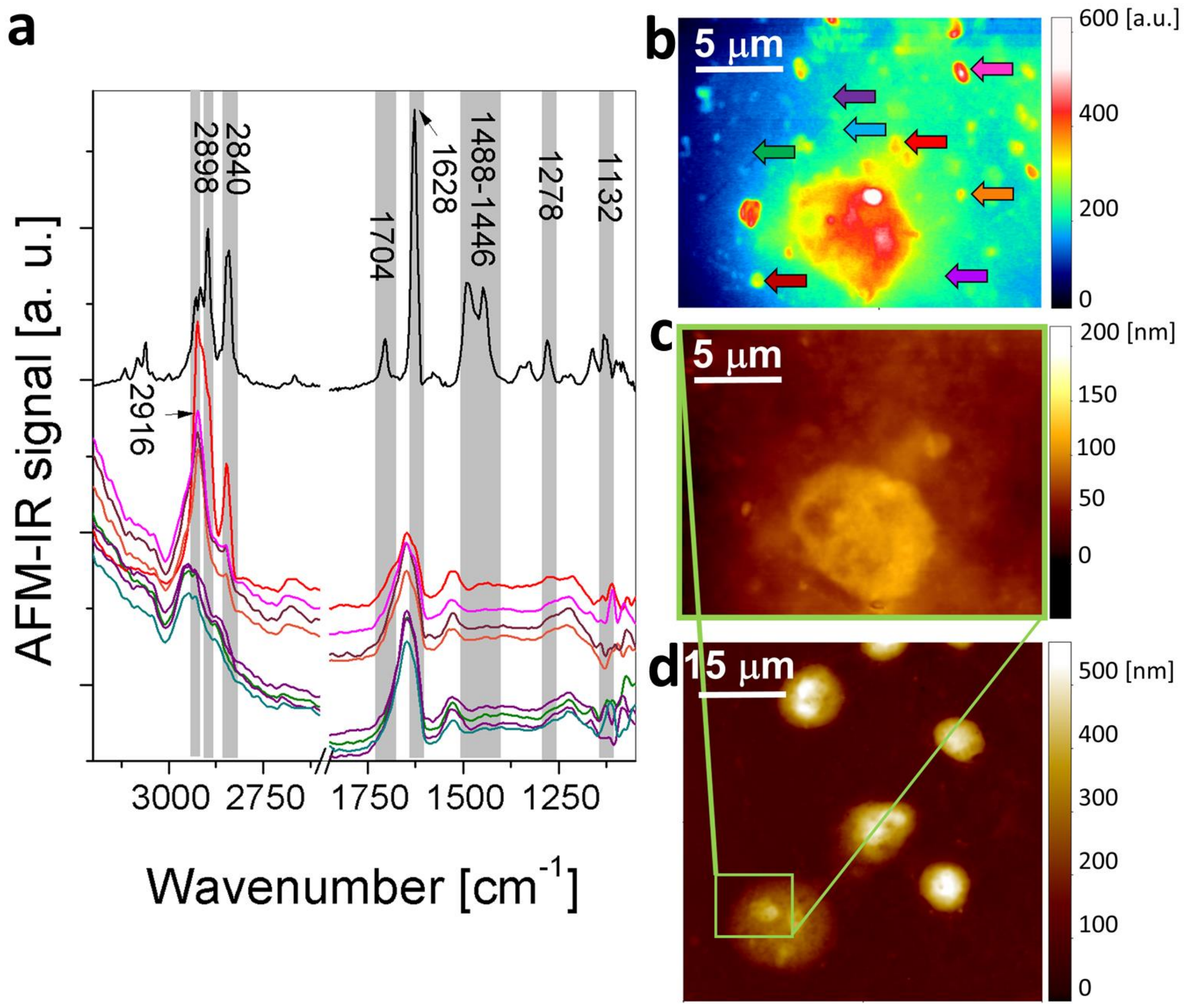

Supplementary Figure S14 Infrared nanospectroscopy imaging of cellular nuclei treated with Pt-103: AFM-a) IR spectra collected from the places marked by arrows; on b) color of each arrow corresponds to color of each spectrum, b) the distribution of infrared absorption at 2916 cm^{-1}, c) and d) two AFM topographies of isolated cellular nuclei, zoomed area c) corresponds to b).

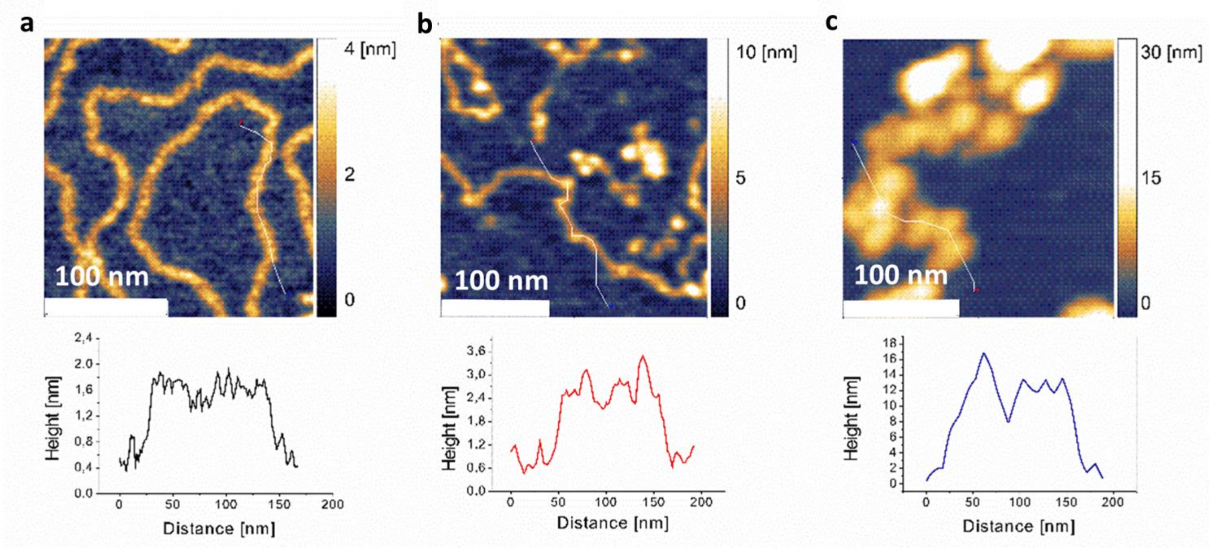

Supplementary Figure S15 AFM studies of interaction between DNA and Pt-103: a) control DNA fixed on mica with profile extracted along line, b) DNA incubated with 200 μM of Pt-103 for 2 h and c) for 12 hours together with profiles extracted along lines visible on topographies indicating globular features – condensed DNA upon interaction with the drug.

S13 Supplementary References:

1. IAEA (2001) Cytogenetic Analysis for Radiation Dose Assessment. *Tech. Reports Ser.*, **405**.

2. Unger,M. and Marcott,C. (2017) Recent Advances and Applications of Nanoscale Infrared Spectroscopy and Imaging. In *Encyclopedia of Analytical Chemistry*. John Wiley & Sons, Ltd, pp. 1–26.

3. Dazzi,A., Prater,C.B., Hu,Q., Chase,D.B., Rabolt,J.F. and Marcott,C. (2012) AFM–IR: Combining Atomic Force Microscopy and Infrared Spectroscopy for Nanoscale Chemical Characterization. *Appl. Spectrosc.*, **66**, 1365–1384.

4. Lahiri,B., Holland,G. and Centrone,A. (2013) Chemical imaging beyond the diffraction limit: Experimental validation of the PTIR technique. *Small*, **9**, 439–445.

5. Janik,E., Bednarska,J., Zubik,M., Puzio,M., Luchowski,R., Grudzinski,W., Mazur,R., Garstka,M., Maksymiec,W., Kulik,A., *et al.* (2013) Molecular Architecture of Plant Thylakoids under Physiological and Light Stress Conditions : A Study of Lipid – Light-Harvesting Complex II Model Membranes. *Plant Cell*, **25**, 2155–2170.

6. Heymann,J.B., Moller,C. and Muller,D.J. (2002) Sampling effects influence heights measured with atomic force microscopy. *J. Microsc.*, **207**, 43–51.

7. Ruggeri,F.S., Vieweg,S., Cendrowska,U., Longo,G., Chiki,A., Lashuel,H.A. and Dietler,G. (2016) Nanoscale studies link amyloid maturity with polyglutamine diseases onset. *Sci. Rep.*, **6**, 31155.

8. Dazzi,A. and Prater,C.B. (2017) AFM-IR: Technology and applications in nanoscale infrared spectroscopy and chemical imaging. *Chem. Rev.*, **117**, 5146–5173.

9. Dazzi,A., Glotin,F. and Carminati,R. (2010) Theory of infrared nanospectroscopy by photothermal induced resonance. *J. Appl. Phys.*, **107**.

10. Ruggeri,F.S., Longo,G., Faggiano,S., Lipiec,E., Pastore,A. and Dietler,G. (2015) Infrared nanospectroscopy characterization of oligomeric and fibrillar aggregates during amyloid formation. *Nat. Commun.*, **6**, 7831.

11. Katzenmeyer,A.M., Holland,G., Kjoller,K. and Centrone,A. (2015) Absorption Spectroscopy and Imaging from the Visible through Mid-Infrared with 20 nm Resolution. *Anal. Chem.*, **87**, 3154–3159.

12. Mantsch,H.H. and Chapman,D. eds. (1996) Infrared Spectroscopy of Biomolecules Wiley–Liss.

13. Dopico,A.M. ed. (2007) Methods in Molecular Biology: Methods in Membreane Lipids Springer.

14. Banyay,M., Sarkar,M. and Graslund,A. (2003) A Library Of Ir Bands of Nucleic Acids In Solution. *Biophys. Chem.*, **104**, 477–488.

15. Socrates,G. (2004) Infrared characteristic group frequencies 3rd ed. John Wiley and Sons, New York.

16. Stuart,B.H. (2004) Infrared Spectroscopy: Fundamentals and Applications John Wiley and Sons, New York.

17. Vila,R., Ponte,I., Collado,M., Arrondo,J.L.R. and Suau,P. (2001) Induction of secondary structure in a COOH-terminal peptide of histone H1 by interaction with the DNA: An infrared spectroscopy study. *J. Biol. Chem.*, **276**, 30898–30903.

18. Froehlich,E., Mandeville,J.S., Weinert,C.M., Kreplak,L. and Tajmir-Riahi,H.A. (2011) Bundling and aggregation of DNA by cationic dendrimers. *Biomacromolecules*, **12**, 511–517.

19. Marty,R., N'soukpoé-Kossi,C.N., Charbonneau,D., Weinert,C.M., Kreplak,L. and Tajmir-Riahi,H.A. (2009) Structural analysis of DNA complexation with cationic lipids. *Nucleic Acids Res.*, **37**, 849–857.

20. Banyay,M. and Gräslund,A. (2002) Structural effects of cytosine methylation on DNA sugar pucker studied by FTIR. *J. Mol. Biol.*, **324**, 667–676.

21. Whelan,D.R., Bambery,K.R., Heraud,P., Tobin,M.J., Diem,M., McNaughton,D. and Wood,B.R. (2011) Monitoring the reversible B to A-like transition of DNA in eukaryotic cells using Fourier transform infrared spectroscopy. *Nucleic Acids Res.*, **39**, 5439–5448.

22. Wood,B.R. (2016) The importance of hydration and DNA conformation in interpreting infrared spectra of cells and tissues. *Chem. Soc. Rev.*, **45**, 1980–1998.

23. Banyay,M. and Gräslund,A. (2002) Structural Effects of Cytosine Methylation on DNA Sugar Pucker Studied by FTIR. *J. Mol. Biol.*, **324**, 667–676.

24. Han,C., Srivastava,A.K., Cui,T., Wang,Q. and Wani,A.A. (2015) Differential DNA lesion formation and repair in heterochromatin and euchromatin Summary : Our findings demonstrated that DNA lesions that induce major helix distortion only form in euchromatin , and SIRT1 plays a critical role in restricting the formation. 10.1093/carcin/bgv247.

25. Roque,A., Ponte,I., Arrondo,J.L.R. and Suau,P. (2008) Phosphorylation of the carboxy-terminal domain of histone H1: Effects on secondary structure and DNA condensation. *Nucleic Acids Res.*, **36**, 4719–4726.

26. Dreveny,I., Deeves,S.E., Fulton,J., Yue,B., Messmer,M., Bhattacharya,A., Collins,H.M. and Heery,D.M. (2014) The double PHD finger domain of MOZ/MYST3 induces alpha-helical structure of the histone H3 tail to facilitate acetylation and methylation sampling and modification. *Nucleic Acids Res.*, **42**, 822–835.

27. Letellier,R., Ghomi,M. and Taillandier,E. (1987) Interpretation of DNA vibration modes. II--The adenosine and thymidine residues involved in oligonucleotides and

polynucleotides. *J. Biomol. Struct. Dyn.*, **4**, 663–83.

28. Matthäus,C., Boydston-White,S., Miljkovi,M., Romeo,M. and Diem,M. (2006) Raman and Infrared Microspectral Imaging of Mitotic Cells. *Appl Spectrosc.*, **60**, 1–8.

29. Liquier,J. and Taillandier,E. (1996) Infrared spectroscopy of nucleic acids. In Mantsch,H.H., Chapman,D. (eds), *Infrared Spectroscopy of Biomolecules*. Wiley-Liss, New York, pp. 131–158.

30. Bolzer,A., Kreth,G., Solovei,I., Koehler,D., Saracoglu,K., Fauth,C., M??ller,S., Eils,R., Cremer,C., Speicher,M.R., *et al.* (2005) Three-dimensional maps of all chromosomes in human male fibroblast nuclei and prometaphase rosettes. *PLoS Biol.*, **3**, 0826–0842.

31. Moyzis,R.K., Torney,D.C., Meyne,J., Buckingham,J.M., Wu,J.R., Burks,C., Sirotkin,K.M. and Goad,W.B. (1989) The distribution of interspersed repetitive DNA sequences in the human genome. *Genomics*, **4**, 273–289.

32. Korenberg,J.R. and Rykowski,M.C. (1988) Human genome organization: Alu, LINES, and the molecular structure of metaphase chromosome bands. *Cell*, **53**, 391–400.

33. Bártová,E., Krejčí,J., Harničarová,A., Galiová,G. and Kozubek,S. (2008) Histone Modifications and Nuclear Architecture: A Review. *J. Histochem. Cytochem.*, **56**, 711–721.

34. International Human Genome Sequencing Consortium (2004) Finishing the euchromatic sequence of the human genome. *Nature*, **431**, 931–45.

35. Strachan,T. and Read,A. (2010) Organization of the human genome. In *Human Molecular Genetics*. Garland Science Taylor & Francis Group, pp. 255–296.

36. Yasukochi,Y., Maruyama,O., Mahajan,M.C., Padden,C., Euskirchen,G.M. and Schulz,V. (2010) X chromosome-wide analyses of genomic DNA methylation states and gene expression in male and female neutrophils. *Proc Natl Acad Sci U S A*, **107**, 3704–3709.

37. Zhao,Y. and Truhlar,D.G. (2008) The M06 suite of density functionals for main group thermochemistry, thermochemical kinetics, noncovalent interactions, excited states, and transition elements: Two new functionals and systematic testing of four M06-class functionals and 12 other function. *Theor. Chem. Acc.*, **120**, 215–241.

38. Dunning Jr,T.H. (1989) Gaussian basis sets for use in correlated molecular calculations. I. The atoms boron through neon and hydrogen. *J. Chem. Phys.*, **90**, 1007.

39. Frisch,M.J., Trucks,G.W., Schlegel,H.B., Scuseria,G.E., Robb,M.A., Cheeseman,J.R., Scalmani,G., Barone,V., Mennucci,B., Petersson,G.A., *et al.* (2009) Gaussian 09, Revision D.01. *Gaussian Inc.*, 10.1159/000348293.

40. Junaid,A., Moon,M.C., Harding,G.E.J., Zahradka,P., Kim,H., Lee,H., Jun,J., Oh,Y., Choi,S., Kim,H., *et al.* (2007) Osteopontin localizes to the nucleus of 293 cells and associates with polo-like kinase-1. *Am. J. Physiol. Cell Physiol.*, **292**, C919-26.

41. Lipiec,E., Wood,B.R., Kulik,A., Kwiatek,W.M. and Dietler,G. (2018) Nanoscale Investigation into the Cellular Response of Glioblastoma Cells Exposed to Protons. *Anal. Chem.*, **90**, 7644–7650.
